# Comprehensive *in silico* modeling of the rice plant PRR Xa21 and its interaction with RaxX21-sY and OsSERK2†

**DOI:** 10.1039/d0ra01396j

**Published:** 2020-04-21

**Authors:** M. H. M. Mubassir, M. Abu Naser, Mohd Firdaus Abdul-Wahab, Tanvir Jawad, Raghib Ishraq Alvy, Salehhuddin Hamdan

**Affiliations:** Department of Mathematics and Natural Sciences, BRAC University 66 Mohakhali Dhaka-1212 Bangladesh mhmmubassir@bracu.ac.bd; Faculty Bioscience and Medical Engineering, Universiti Teknologi Malaysia 81310 Johor Bahru Johor Malaysia saleh65@utm.my

## Abstract

The first layer of defense that plants deploy to ward off a microbial invasion comes in the form of pattern-triggered immunity (PTI), which is initiated when the pattern-recognition receptors (PRRs) bind with the pathogen-associated molecular patterns (PAMPs) and co-receptor proteins, and transmit a defense signal. Although several plant PRRs have been discovered, very few of them have been fully characterized, and their functional parameters assessed. In this study, the 3D-model prediction of an entire plant PRR protein, Xa21, was done by implementing multiple *in silico* modeling techniques. Subsequently, the PAMP RaxX21-sY (sulphated RaxX21) and leucine-rich repeat (LRR) domain of the co-receptor OsSERK2 were docked with the LRR domain of Xa21. The docked complex of these three proteins formed a heterodimer that closely resembles the other crystallographic PTI complexes available. Molecular dynamics simulations and MM/PBSA calculations were applied for an in-depth analysis of the interactions between Xa21 LRR, RaxX21-sY, and OsSERK2 LRR. Arg230 and Arg185 from Xa21 LRR, Val2 and Lys15 from RaxX21-sY and Lys164 from OsSERK2 LRR were found to be the prominent residues which might contribute significantly in the formation of a heterodimer during the PTI process mediated by Xa21. Additionally, RaxX21-sY interacted much more favorably with Xa21 LRR in the presence of OsSERK2 LRR in the complex, which substantiates the necessity of the co-receptor in Xa21 mediated PTI to recognize the PAMP RaxX21-sY. However, the free energy binding calculation reveals the favorability of a heterodimer formation of PRR Xa21 and co-receptor OsSERK2 without the presence of PAMP RaxX21-sY, which validate the previous lab result.

## Abstract 2

This study exhausts bioinformatics tools to acquire the entire multi-domain rice Xa21 protein structure and analyzes its interactions with its PAMP RaxX21-sY and co-receptor OsSERK2.

## Graphical abstract

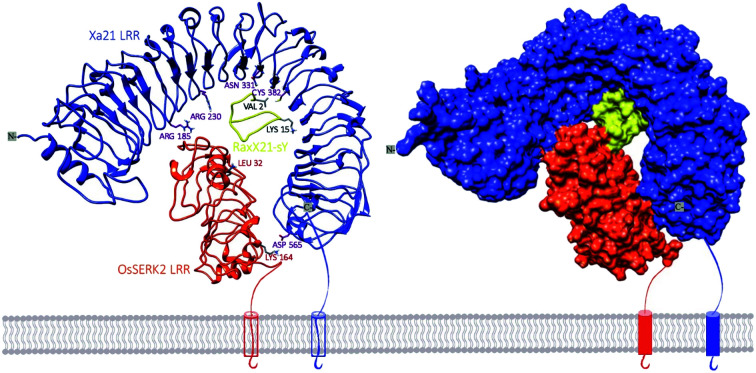

## Introduction

Plants employ a two layered defense mechanism to confront bacterial and fungal pathogens. The first layer of defense is termed pattern-triggered immunity (PTI), which is the focus of the current study; and the second layer is called effector triggered immunity (ETI).^1–3^ PTI is triggered upon the recognition of a conserved microbial signature by the host leucine-rich repeat (LRR) domain of pattern recognition receptors (PRRs).^4^

Based on their location in the cell, PRRs are classified as membrane-bound PRRs and cytoplasmic PRRs. Subsequently, membrane bound plant PRRs which confers innate immunity are classified into receptor-like proteins (RLPs), which do not have a kinase domain; and receptor-like kinases (RLKs), which have a well-defined kinase domain.^5–9^ Plants have a greater number of both RLPs and RLKs compared to animals^10^ and there are more RLKs than RLPs. While the rice plant has 640 RLKs, the *Arabidopsis* plant only has around 410 RLKs.^10–12^ There are four vital roles played by RLKs, which are sensing the extracellular signals such as peptides or ligands, activate receptor proteins, carry out downstream signals, and anticipating protein interaction sites and make sizable signaling networks.^13^

PRR in *Arabidopsis* such as flagellin-sensitive 2 (FLS2) was well characterized experimentally^14^ while the tertiary structure of PRR protein in rice known as Xa21, is yet to be resolved. FLS2 and Xa21 both fall under the protein XII sub-family (leucine-rich repeat-receptor kinase, LRR-RK XII). Sub-family XII is one of the most expanded of LRR-RK families in rice, which encode mostly for PRRs or PRR-associated RKs.^15^ Xa21 also shows enormous similarity in defense signaling both with animal toll-like receptors (TLRs) and EF-Tu receptors, besides FLS2.^7,16,17^

Due to the presence of Xa21, rice plants show robust resistance to bacterial leaf blight (BLB) pathogen *Xanthomonas oryzae* pv. *oryzae* (*Xoo*).^18,19^ BLB is regarded as a major disease in rice^20^ which can cause yield loss of up to 70%.^21^ The best control and by far the most cost effective approach has been the usage of resistant cultivars.^22,23^ Xa21 has 23 leucine rich repeats in its LRR domain, one transmembrane (TM) domain, one juxtamembrane (JM) domain and one kinase domain.^24^ The LRR domain, which is localized outside of the plant membrane, binds to the bacterial peptide, RaxX21 secreted by *Xoo*. During bacterial secretion, the RaxX21 becomes sulfated in the tyrosine region (RaxX21-sY).^25^ This, in turn, imparts increased stability to the peptide, leading to the initiation of the subsequent task of binding to Xa21 and initiating a signal.^26^ Consequently, this signal passes through the TM domain and JM domain to the kinase domain, activating the PTI which leads to the association of the co-receptor protein OsSERK2 with Xa21, triggering the plant defense mechanism.^27^ Plants without PRR Xa21 shows susceptibility to the RaxX21-sY generating *Xoo* strain, and interestingly plants with PRR Xa21 cannot recognize *Xoo* strain without the presence of RaxX21-sY.^25,28–30^

For other similar cases like FLS2, its LRR domain binds to flg22 which is a 22 residue epitope situated at the N terminus of flagellin from Gram-negative bacteria with the help of coreceptor BAK1 to trigger PTI.^14^ For BRI1, another well characterized PTI complex, its LRR domain binds with the co-receptor SERK1 and forms a heterodimer where the PAMP brassinolide acts as a molecular glue.^31^ Besides activating the defense mechanism, several other PRRs are also involved in controlling the growth and development of the plant, such as HAESA,^32^ formerly known as RLK5, which recognizes the IDA to control the floral organ abscission.

Only about 2% of all membrane bound proteins have been crystallized and the information added to the protein data bank (PDB).^33^ Also, no crystallographic structure of entire plant PRRs are available in the PDB to date, only partial structures are available (such as LRRs or kinase domains). As Xa21 is a membrane bound PRR with a complicated four-component configuration, no structure of Xa21 has yet been determined experimentally, therefore *in silico* modeling approaches were implemented to predict a structural model of Xa21.

Furthermore, the modeled LRR domain of Xa21 was docked with PAMP RaxX21-sY and co-receptor OsSERK2 LRR, and molecular dynamics (MD) simulations and MM/PBSA free energy binding calculations were performed to get a better understanding on binding mechanism of the PAMP RaxX21-sY, co-receptor OsSERK2 and the PRR Xa21. Besides, the MD simulation and MM/PBSA calculation of different complexes of the Xa21 provides important insight on the contribution of the PAMP RaxX21-sY and co-receptor OsSERK2 in the formation of the heterodimer complex for PTI. We believe that, the outcome of this study will not only contribute significantly towards the understanding of the structural details and functional aspects of PRR Xa21, but also its interaction with PAMP RaxX21-sY and co-receptor OsSERK2 LRR, as well as their individual contributions in Xa21 mediated immunity.

## Materials and methods

### Sequence based analysis and delineation of the domain boundary

Amino acid (AA) sequence of the target Xa21 protein was retrieved from Uniprot KB with the accession number Q40640 and the sequence was originally reported to the database by Song *et al.* in 1995.^24^ The ProtParam tool was employed to analyze the primary structure and to predict the physio-chemical properties,^34^ whereas the secondary structure was predicted using PSIPRED (V3.3)^35^ and SOPMA.^36^ To identify the conserved region of the sequence the ConSurf tool^37^ was used. To investigate the domain architecture InterPro,^38^ SMART,^39^ lrrfinder.com^40^ and HHrepID^41^ tools were used. PREDICT PROTEIN,^42^ TMHMM,^43^ MEMSAT-SVM^44^ and SOSUI^45^ tools were used for predicting the transmembrane region.

### Single template modeling

NCBI BLASTP^46^ and HHpred^47^ analysis for homology detection of Xa21 AA sequence was carried out against the Protein Data Bank (PDB) using default parameter values to search for suitable templates for Xa21. Then different single template modeling approaches were implemented (Table S1A†) such as – Modeller 9.15,^48^ 3D-JIGSAW,^49^ CPHmodel 3.2,^50^ Geno3D,^51^ PRC,^52^ Prospect 2,^53^ pGenTHREADER,^54^ FFAS-3D,^55^ FFAS03,^56^ SP3,^57^ Sparks-X,^58^ Swiss-Model,^59^ MUSTER^60^ and wdPPAS.^61^

### Multiple template modeling

Multiple sequence alignment (MSA) of the top five templates (according to lowest e-value resulted from BLASTP) and the target sequence was carried out using the Praline tool.^62^ The result illustrated a comparative analysis of the template sequence against the target sequence and helped visualize the template sequence coverage of the target sequence. MSA (Fig. S1a†) showed that the templates only covered either the LRR region or the kinase domain of Xa21. The domain boundary was then set to Xa21 N-terminal, LRR (AA 1–634), Xa21 kinase, JM, TM charged1 (c1), TM charged2 (c2) and C-terminal (AA 635–1025) regions to see if any of the templates covered more than one domain of Xa21. Then a second MSA was performed using the entire sequence of Xa21 containing all the domains with top five complementary template sequences for the LRR domain, while a third MSA was carried out using the entire target sequence of Xa21 against top five complementary template sequences for the kinase domain which resulted from the latest blast (Table S2A†).

From the second and third MSAs (Fig. S1b and c†), it was evident that there was no overlap of the kinase, JM, and TM regions with the LRR template; while some of the kinase domain templates had overlapping regions with the JM domain region of the Xa21 protein. The results also showed that the N-terminal region (AA 1–26) and C-terminal region (AA 1010–1025) had no corresponding aligned regions and played no part in the PAMP or co-receptor binding. Therefore, these two regions were omitted from the target sequence. Also, from the results of the second and third MSAs it was evident that there was no complementary sequence for the TM region. Then NCBI BLASTP was again carried out for Xa21 TM, c1 and c2 (AA 651–707) regions to search for a suitable template for the TM region. But no complementary template was found.

Again, from the third MSA (Fig. S1c†), it was observed that the kinase domain showed overlaps with the JM domain. To find out templates for the kinase and JM domains, NCBI BLASTP was carried out using respective query sequences and the top five templates were selected to be used in multiple template modeling. In all cases, the same structure with different PDB ID was avoided.

**Fig. 1:**
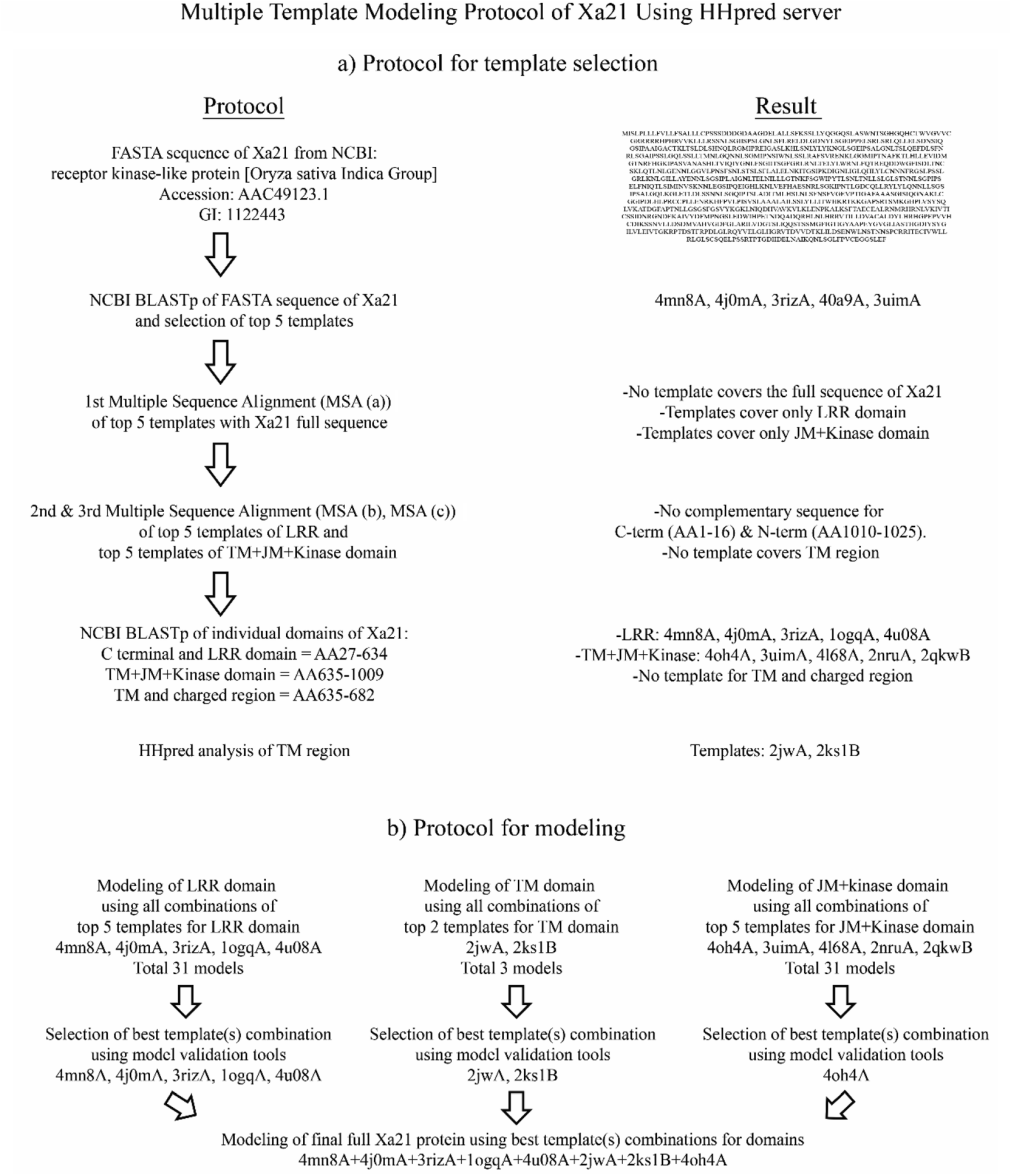
Multiple template modeling protocol of Xa21 using the HHpred server.

For LRR domain modeling with the HHpred server, the top five templates were selected from BLASTP results (Table S2A†). Five templates were selected because of the HHpred server's recommended use of a maximum of five templates for each domain. All combinations for the 5 templates were considered for building models of the LRR domain (Table S1B†). According to the equation below a total of 31 models were built. The same procedure was followed for kinase and JM domain modeling (Fig. 1).
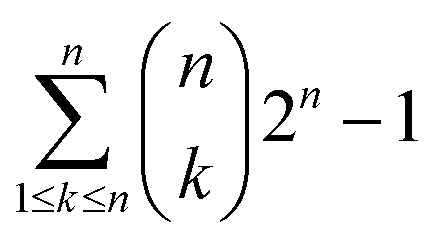
where, *n* – total number of templates (which is always five in this case); *k* – number of templates being used for a particular combination.

As NCBI BLASTP could not find any template sequences for TM region, the HHpred server was used instead and two templates were found with reasonable e values (Table S2B†). Hence for modeling the TM region, the combination of these two templates (making it a total of three) was used.

For modeling the final Xa21 protein with the HHpred server, the individual domain models modeled by the HHpred server (which were evaluated to be the best) were taken into consideration. Finally, modeling was done using the best template/combination of templates of these domains (Fig. 1).

Other multiple template modeling tools, having different modeling principles, such as Phyre2 intensive modeling,^63^ Raptor-X,^64^ I-TASSER^65^ and AIDA^66^ were also used to model Xa21; to ascertain that the final model chosen for further studies was the best possible one. All these tools are automated protein modeling servers where Phyre2 is a homology-based prediction tool and uses Poing (an *ab initio* tool) to predict small fragments where homology is missing, Raptor-X is based on threading, I-TASSER is based on both threading and *ab initio*, and AIDA is a homology based multiple template modeling server.

### Structure validation

To analyze the geometry, structural consistency and reliability of the predicted structures, multiple tools were employed. To evaluate the non-bonded interactions between different types of atoms, ERRAT^67^ was used, while Verify-3D^68^ was employed for assessing the compatibility of the atomic models by subjecting them to their own AA sequences. For geometrical consistency analysis, Ramachandran plots generated by PROCHECK^69^ were assessed. The quality of these proteins were also visually analyzed using PyMOL.^70^ The quality of the final models was again checked in more detail using ProQ,^71^ ProSA,^72^ VADAR,^73^ and MolProbity.^74^

### Docking of Xa21 LRR with its PAMP RaxX21-sY and co-receptor OsSERK2 LRR

For investigating the interaction of PAMP RaxX21-sY and co-receptor OsSERK2 LRR with its PRR Xa21, only the LRR region of the Xa21 protein was taken into consideration, as only LRR portion of the protein is enough to initiate the defense signal.^32,75–79^ The best LRR structure of Xa21 (Xa21lrr_15) and the RaxX21-sY modeled in our previous study^26^ were energy minimized and equilibrated. A 2 ns NVT equilibration followed by a 1 ns NPT equilibration was performed. The subsequent energy minimized and equilibrated structures were used as initial protein structures for docking using the protein–protein docking tool ZDOCK.^80^ The top 10 predictions were obtained from docking results and the best structure, according to the binding similarity with other crystallographic structures, was carefully selected. Following the same protocol OsSERK2 LRR (PDB ID: 4q3g) was docked with the complex of Xa21 and RaxX21-sY, and complex 1 (Xa21 LRR with its PAMP RaxX21-sY and co-receptor OsSERK2 LRR) was prepared for a MD simulation. The 27^th^ AA glycine of Xa21 LRR was considered to be the 1^st^ AA and the results of the prediction were analyzed accordingly. To get an idea about the contribution of co-receptor OsSERK2 and PAMP RaxX21-sY, two other complexes were made from complex 1 which were complex 2 (by removing PAMP RaxX21-sY from the complex 1) and complex 3 (by removing co-receptor OsSERK2 from the complex 1).

### MD simulation of Xa21 LRR, RaxX21-sY and OsSERK2 LRR complexes

All three complexes were subjected to MD simulation with GROMACS^81^ software suite (version 5.1). GROMOS 54a7 (ref. 82) united force field was employed for the simulation process, using water as a solvent. For solvation, cubic boxes were made with a minimum distance of 1 Å between the surfaces and edges of the complexes and were solvated with the SPC water model.^83^ After neutralizing the systems with the Genion tool, energy minimization was done for all the systems. Then the systems were equilibrated for 2 ns NVT ensemble followed by 1 ns NPT ensemble maintaining a constant 300 K temperature and 1 atm pressure, respectively. Finally, a 100 ns MD simulation was carried out for each complex. Particle Mesh Ewald (PME) method was applied to treat the long-range electrostatic interactions. Root mean square fluctuations (RMSF) were calculated using the GROMACS tool to monitor the prominent residues of the proteins inside the complexes.

### Analysis of binding mode of Xa21 LRR with RaxX21-sY and OsSERK2 LRR

Binding mode of Xa21 LRR, RaxX21-sY and OsSERK2 LRR complex was assessed using UCSF Chimera.^84^ Besides, the complex was compared with other similar crystallographic structures obtained from the protein data bank, consisting of – the crystal complex of FLS2 LRR-flg22-Bak1 (PDB ID: 4mn8), PEPR1-AtPep (PDB ID: 5gr8), BRI1-brassinolide-BAK1 (PDB ID: 4lsx) and HAESA-IDA-SERK1 (PDB ID: 5iyx).

For analyzing the individual residue contribution during binding of Xa21 LRR with RaxX21-sY and OsSERK2 LRR, MM/PBSA and protein interaction calculator (PIC) were employed. MM/PBSA is a post-processing method where the free energy of a state is determined from the interior energy (MM) of the residues and its connection with an understandable portrayal of solvent (PBSA).^85^ For MM/PBSA calculation, the g_mmpbsa^86^ tool was used which is developed by using GROMACS and ABPS tools. g_mmpbsa computes binding energy of bio molecular affiliations like protein–protein, protein–ligand, protein–DNA *etc.* It gives the distinctive segment of energy term in independent record with the goal of having either MM, PB and SA energy esteems or all of them. Moreover, in the g_mmpbsa tool, two types of python scripts (MmPbSaStat.py and MmPbSaDecomp.py) were used to calculate the average binding energy and its standard deviation/error and the final contribution energy of each residue from individual energetic terms.

For the MM/PBSA calculation, complex 1 was divided into three different sub-complexes which are complex 1a, 1b and 1c as the tool can only calculate the binding energy between two proteins. Complex 1a is comprised of Xa21 LRR and RaxX21-sY, complex 1b contains Xa21 LRR and OsSERK2 LRR and complex 1c consists of RaxX21-sY and OsSERK2 LRR. Similar protocols were followed for MM/PBSA calculation in case of complex 2 and complex 3 as well.

By using PyMOL^87^ and UCSF Chimera^84^ all the structures and the intermolecular interactions among Xa21 LRR, RaxX21-sY and OsSERK2 LRR were visualized. H-bonds, hydrophobic interactions, ionic interactions, aromatic interactions and cation–pi interactions between these proteins were measured using the tool protein interaction calculator (PIC). All these analyses were done for each of the docked complexes both before and after the simulations.

## Results and discussion

### Sequence based analysis and delineation of the domain boundary

The primary structure of Xa21 was analyzed using the ProtParam tool. Xa21 is a relatively large protein containing 1025 amino acids. A theoretical estimate of the molecular weight of Xa21 is 111.34 kD which is lower than that of the experimentally measured value. The isoelectric point (pI) of Xa21 was computed to be 7.35, suggesting that the protein is slightly alkaline. The aliphatic index (109.69) and the instability index (32.79) of Xa21 suggested that the protein is thermostable. Finally, the GRAVY value (0.049) indicated that Xa21 is hydrophobic in nature. Since Xa21 has zero net charge and is hydrophobic in nature, Xa21 is not likely to be an intrinsically disordered protein.

SOPMA was used to make the following predictions about the secondary structure – alpha helix 35.12%, extended strand 18.15%, beta turn 8.49% and random coil 38.24%, while no 3_10_ helix, pi helix, beta-bridge, bend region and ambiguous states were found. A detailed observation of the SOPMA results showed that the transmembrane region (658–672) was most likely predicted as the alpha helix. The secondary structure predicted by PSIPRED (Fig. S2†) was consistent with that of SOPMA.

The ConSurf tool was used to predict the conserved regions of Xa21. On the basis of the calculations which portrayed a sufficiently low evolutionary rate, the kinase domain (708–799) was predicted to be the conserved region (Fig. S3a†). The other regions such as N-terminal, C-terminal, TM domain and LRR domain were classified as variable regions as those regions had a relatively higher evolutionary rate.

To perform functional annotation of the Xa21 protein sequence, the InterPro tool was used. InterPro reported two LRR domains, one protein kinase like domain and one concanavalin A-like lectin/glucanase domain (Fig. S3b†). It also detected a protein kinase ATP binding site and a serine/threonine–protein kinase active site. The detected LRR domain, kinase domain and two sites are very relevant to Xa21's function. Though the role of concanavalin A-like lectin/glucanase domain in Xa21 is not clear, it is suspected to serve as a carbohydrate receptor. The SMART tool was used to explore the domain architecture of Xa21 and the search revealed that Xa21 is composed of 12 LRRs in its domain, one transmembrane domain and one kinase domain (Fig. S3c†). To further investigate the LRR region, lrrfinder and HHrepID tools were used. Lrrfinder detected 13 significant hits for LRR domain and HHrepID predicted 23 LRRs in a domain (Table S3†), which is composed of 24 AA (Fig. S3d†). Among all LRR domain predictions, the HHrepID prediction matched with previous findings by Song *et al.*, 1995.^88^

The transmembrane region predicted by Predict Protein was from AA 656 to 675 whereas TMHMM and SOSUI predicted AA 652 to 674, and MEMSAT-SVM predicted AA 653–675 residues of Xa21 (Fig. S3e†). These predictions were similar to previous findings by Song *et al.*, 1995.^24^ Also, the resulted LRR domain has a consensus sequence LxxLxxLxLxxNxLSGxIPxxLGx which is very unique to plant LRRs which creates a twisted or helical horseshoe like structure.^31^

### Single template modeling

NCBI BLASTP analysis of the 1025 AA sequence of Xa21 protein resulted in several templates according to the query coverage, e-value and identity. Among the templates, 4mnA (FLS2 ectodomain) has the highest score (340) with the lowest e-value (8e-103). The suggested template sequence has 36.65% identity and 58% coverage with the query sequence. HHpred also reported 4mn8A to be the best template for modeling Xa21 with an e-value of 6.6 × 10^−42^ and a maximum score of 425.3.

Default parameters were used for modeling Xa21 using Modeller (version 9.15). It produced five of the best models and the model with the lowest DOPE score (−91 944.44531) was taken under consideration for further validation. Other homology modeling tools such as 3D-JIGSAW, CPHmodel 3.2, Swiss-Model and Geno3D failed to produce a working model of the entire protein containing all the amino acids of Xa21. Another attempt was also made to build a model of Xa21 using a local meta-threading server, LOMETS. LOMETS server uses ten locally installed threading programs such as FFAS-3D, MUSTER, pGenTHREADER, PPAS, PRC, PROSPECT2, SP3 and SPARKS-X, and none of the programs were able to model the entire protein of Xa21 (Fig. S4†).

### Multiple template modeling

Through the visual screening using PyMOL and the use of different validation servers the quality of the different protein structures was assessed (Table S4†) and it was evident that model Xa21lrr_15 built from the five templates (4mn8A + 4u08A + 4j0mA + 3rgzA + 1ogqA) is the best LRR domain model among all the 31 models generated using the HHpred server. The ERRAT score of 63.167 suggested that the generated model is robust. The Verify-3D result turned up a 100% which indicates excellent compatibility of the model with its own AA sequence. Ramachandran plot analysis from Procheck showed that 98.2% (70.6 + 26.6 + 1) of residues are in the allowed region, which indicates that the model is of very high quality.

For the kinase domain containing JM domain and C terminal, model Xa21k_1 built from only one template (4oh4A) gave the maximum ERRAT score. Verify-3D result also showed significant compatibility (84.71%) of the model with its own AA sequence. The Ramachandran plot also showed that 99.7% (90.6 + 7.7 + 1.4) of the residues were in the allowed region which dictates that the generated model is of very high quality. For TM region containing C1 and C2 regions, model Xa21c12tm_3 built from templates 2jwaA and 2ks1B gave the best ERRAT score (81.579) and 100% residues are also in the allowed region of the Ramachandran plot (Table S3†). All these eight templates (4mn8A + 4j0mA + 3rizA + 1ogqA + 4u08A + 2jwA + 2ks1B + 4oh4A) were determined to be the best for all individual domains and so were selected for the final modeling of Xa21 (AA 27–1009) using the HHpred server.

In addition to the HHpred server, Phyre2 suite was also used to model the Xa21 protein. In Phyre2 intensive model, 8 templates were fed into Phyre2 and heuristics was applied to maximize confidence, percent identity and alignment coverage. A total of 91% of residues were modeled at >90% confidence and 90 residues were modeled by a combination of homology and *ab initio* method. Since it has >90% confidence level and high identity, we assumed that the predicted model is fairly accurate.

The Raptor-X tool predicted two domains (modeling unit) in multiple template modeling of Xa21. Domain1 is of LRR region and domain2 is of TM, JM and kinase domain portions. In the case of domain-1 modeling, 4mn8A was selected as the template and for modeling domain-2 3tl8A, 4oa2A and 4oa6A were selected as templates.

Top ten threading templates were selected from the tool I-TASSER, based on the highest *Z*-score of each threading alignment of LOMETS from thousands of threading alignments. Five models were generated according to low C-score values and finally the model with the lowest C-score (−2.05) was selected for validation.

AIDA predicted two domains – domain1 and domain2, for Xa21 protein modeling and domain2 was further divided into domain2.1 and domain2.2. 4mn8A template was used for modeling domain1, 2jwaA for modeling domain2.1 and 4l68A for domain2.2. Finally, all the domains were assembled to completely model the Xa21 protein.

### Structure validation of models for single template modeling approach

Screening (Fig. S4†) of the Xa21 proteins generated by single template modeling approach gave us an idea about the multi-domain protein modeling capability of these tools. Among single template-based tools, 3D-JIGSAW, CPHmodel 3.2, Geno3D and Swiss-Model could not generate the entire tertiary structure. These servers only generated the 3D structure of the LRR domain of Xa21. Similarly, PRC, PROSPECT2, FFAS-3D, FFAS03, SP3, MUSTER, pGenTHREADER and wdPPAS failed to predict 3D structures of the TM, JM and kinase domain regions (Fig. S4†). Although, Modeller (version 9.15) and Sparks-X generated the entire protein's 3D structures, patchy unpredicted regions were present in the models. All these incomplete 3D models of Xa21 were discarded and no further investigation was done on these incomplete structures.

### Structure validation of models for multiple template modeling approach

**Fig. 2:**
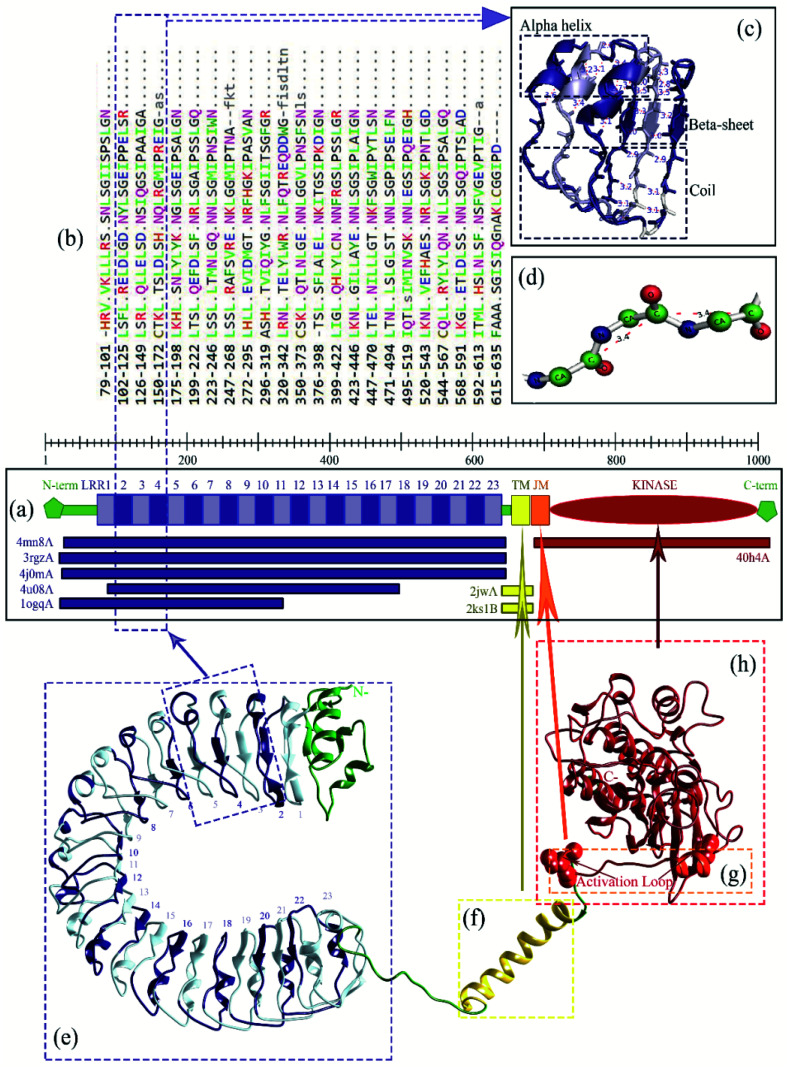
(a) Template alignment and position for 3D modling of Xa21 using the HHpred server; (b) MSA of 23 LRR domains of Xa21; (c) cartoon structure of three LRRs; (d) ball and stick representation of one beta sheet of Xa21 LRR showing the distance between two C-alpha atoms; (e) LRR domain; (f) TM domain; (g) JM domain; and (h) kinase domain of the predicted Xa21 3D structure.

All the multiple template modeling tools modeled 1025 residues of Xa21 (Fig. S5†). For AIDA and I-TASSER, the structure for the LRR domain is moderately consistent with their respective chosen domain templates. Raptor-X and Phyre2 intensive modeling generated LRR and kinase domains which did show moderate consistency but the TM domain remained as a single thread like structure which is neither consistent with the secondary structure predicted, nor similar to its template structure. Only the HHpred server modeled all the domains of Xa21, where consistency of each domain with its counterpart template looks substantially better (Fig. 2a). Except for the model from I-TASSER, all multiple template protein models gave ERRAT value greater than 50 (accepted value). In the case of Verify-3D, none of the models reached the threshold value (>80%) except for the model from the HHpred server (87.18) which indicates good AA compatibility with its own 3D structure. PROCHECK Ramachandran plot of the model generated by the HHpred server showed that 99.2% of residues in the model were in the allowed region (Table 1) suggesting that the quality of the model is better than that of the others. The MSA of the LRRs were aligned with the modeled structure (Fig. 2b) for atomic level validation using PyMOL. Based on which, three LRR beta strands were selected (LRR2, LRR3 and LRR4) for analysis. Then the polar contacts in the intra main chain were observed and found to be within a reasonable distance (Fig. 2c). Then the beta strand of LRR2 was depicted as a ball and stick structure, which helped hide its side chain, that the atomic distance between the two consecutive C-alpha atoms could be measured; and it was measured to be 3.4 Å (Fig. 2d), which indicated that the C-alpha atoms on the beta strands were in a good position (∼3.5 Å).

**Table 1:** Structural validation of the Xa21 proteins modeled using different Multiple Template Modeling approaches^a^

Tool	ERRAT	V. 3D (%)	Ramachandran plot summary from PROCHECK (%)			
			MFR	AAR	GAR	DR
HHpred server	67.179	87.18	78.9	18.4	1.9	0.8
AIDA	57.231	78.99	78	18.9	2.1	1.1
I-TASSER	E	72.68	50.9	35.6	8.2	5.3
Phyre2 (intensive)	56.284	67.71	74.9	19.7	3	2.4
Raptor-X	68.553	76.2	78.2	18.6	1.5	1.8

a V. 3D, Verify-3D; MFR, most favored region; AAR, additionally allowed region; GAR, generously allowed region; DR, disallowed region; E, error.

The model generated by the HHpred server was further validated using ProQ, ProSA, VADAR and MolProbity. The ProQ server gave an LG score of 5.172 suggesting a very good quality model (the requirement being a value greater than 4). The MaxSub score from ProQ was 0.315, indicating also a good quality model. All the scores of ProSA (*Z*-score −8.95), VADAR (standard deviation of *χ*_1_ pooled −0.25, mean H-bond energy 0.65, percentage of generously allowed Ω angles −1.6, percentage of packing defects 1.65) and MolProbity (percentage of bad bond length −0.33, percentage of bad bond angles 1.81 and Ramachandran plot outliers 1.1) (Fig. S6†) further supported that the Xa21 model generated by the HHpred server was of good quality.

A closer investigation of the structural differences between the final 3D structure generated by HHpred server and other structures generated by single template modeling approach was carried out. The analysis revealed several gaps at different sites in the model generated by 3D-JIGSAW, Geno3D, PRC, PROSPECT2, FFAS-3D, FFAS03, Sparks-X, pGenTHREADER, wdPPAS tool and Swiss-Model (Fig. S4†), whereas these are absent in the final model generated by the HHpred server (Fig. S5†). Swiss-model was taken into consideration for a detailed breakdown of the sequence alignment used to develop the model, which was provided in great details by the tool. The first structural gap was observed at AA LYS288-ILE289 region, while the second and largest structural gap was found between ASP339 to THR348. The third and fourth structural deviations were observed at ARG422-LEU423 and MET501-ILE502 regions, respectively (Fig. S7a†). To investigate these deficiencies, the model generated by Swiss-model was further analyzed, which revealed that these gaps were consistent with the deviations in the alignment between the model and the template (Fig S7b†). The larger the observed gap, the more was the deviation; which was not the case with the HHpred generated model (Fig. S7c†) and other multiple template modeling tools. This clearly represents the advantages of multiple template modeling over single template modeling of multi-domain large proteins with low target-template sequence similarity. This has been described earlier by Buenavista *et al.*, where they suggested that the model quality can be improved by multiple template modeling in the case of low sequence similarity (<30%) of the protein with its template.^89^ Considering the multi-domain nature of the Xa21 protein, identifying the best template combination for each domain and incorporating this template information to the HHpred server is the best method for modeling this protein.

In the case of plant PRRs, LRRs are usually 22–23 AAs in length that stack onto each other to make the LRR domain.^90^ The LRR domain of Xa21 exhibits the same architecture of 23 LRRs each composed of 22–23 AAs (Table S3†). Again for the LRR domain of Xa21, the predicted horse-shoe shaped structure (Fig. 2e) is common for other LRRs like FLS2, BRI1 and PSKR.^14,31,91^ Although LRR of BRI1, PSKR and RPK2 exhibit island domains to facilitate the binding of the ligands,^31,77,92,93^ no such island domain was observed for Xa21.

Additionally, where LRRs play the key role in the binding of PAMPs, there are very few studies related to the activation of the transmembrane (TM), juxtamembrane (JM) and cytoplasmic kinase domains.^13^ For EFR mediated immunity, the absence of TM domain resulted in the loss of binding capacity to the PAMP elf18, thus proving the necessity of this domain.^94^ The TM of Xa21, which is mainly composed of an alpha helixes (Fig. 2f), has a significant role in transferring defense signals as the lack of this domain results in partial resistance of the rice plants.^75^

The crystal structure of the cytoplasmic kinase domain was solved for BRI1 and BAK1 which revealed the conformation of the domain. Kinase domain of the PRR in most cases is located adjacent to the TM domain, where there is an N-terminal lobe, a nucleotide-binding site, and a C-terminal lobe.^95,96^ Our predicted Xa21 JM and kinase domains show a structural conformation similar to that of BRI1 and BAK1, where the JM domain is mainly composed of alpha helix and the kinase domain has multiple alpha helixes and beta sheets, starting with an N-terminal lobe, a C-terminal lobe, followed by an activation loop (Fig. 2g).

Both BRI1 and BAK1 kinase domains can transphosphorylate each other upon phosphorylation which is assumed to have a regulatory function.^97–99^ For Xa21, upon binding of the PAMP, the kinase domain is being activated *via* the JM domain which results in autophosphorylation and/or transphosphorylation of the other several proteins downstream.^100^ Several key residues of the JM domain were found to play an important role in transphosphorylation to stabilize and activate the PRR Xa21, which are – Ser686, Thr688 and Ser689.^100,101^ In addition, the kinase domain of the PRR Xa21 (Fig. 2h) interacts and transphosphorylates the XB3, a ubiquitin ligase, which is required for the successful activation of pattern triggered immunity in rice plants.^102^ Thr705 of the JM domain of Xa21 was found to be the key residue for autophosphorylation, as replacement of this residue resulted in Xa21 being non-interactive with other essential proteins – XB3, XB10, XB15 and XB24 – required for Xa21 mediated immunity.^103^

### Analysis of binding mode of Xa21 LRR with RaxX21-sY and OsSERK2 LRR

**Fig. 3:**
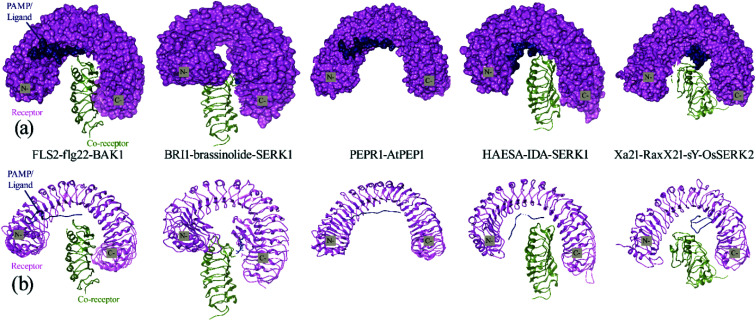
(a) Surface and cartoon structures of LRR-RK signaling complexes: FLS2-flg22-BAK1 (PDB ID 4mn8), BRI1-brassinolide-SERK1 (PDB ID 4lsx), PEPR1-AtPEP1 (PDB ID 5gr8), HAESA-IDA-SERK1 (PDB ID 5iyx) and Xa21-RaxX21-sY-OsSERK2. Receptors are shown in purple surface view, PAMP/ligands are shown in blue surface view and the co-receptors ectodomains are shown in green as cartoon structure; (b) cartoon structures of the FLS2-flg22-BAK1, BRI1-brassinolide-SERK1, PEPR1-AtPEP1, HAESA-IDA-SERK1 and Xa21-RaxX21-sY-OsSERK2.

The docking with Z-Dock shows that RaxX21-sY binds at the lateral concave side of the LRR domain of Xa21 and Xa21 LRR, RaxX21-sY and OsSERK2 LRR form heterodimer (Fig. 3, right most). Other PRRs, such as FLS2, BRI1, PEPR1 and HAESA showed the same binding mechanism with their respective PAMPs. RaxX21-sY binds with Xa21 LRR 7–12, whereas for FLS2, its PAMP flg22 binds at the inner side (concave side) of the outer-domain LRRs 3–18.^14^ In the case of receptor PEPR1, a 23 AA peptide AtPep1 binds at the inner surface of the ectodomain LRRs 4–18 of PEPR1.^104^ Moreover, the PRRs BRI1 and HAESA showed the same binding conformation as Xa21 with their PAMP brassinolide and IDA, respectively (Fig. 3).^31,76,77^

**Fig. 4:**
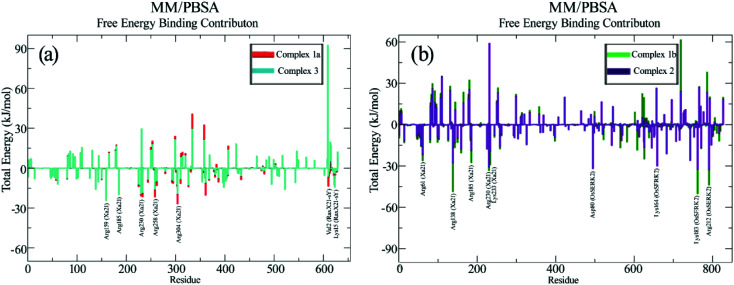
Comparative graphical representation of total binding energy obtained from MM/PBSA calculation between (a) complex 1a and complex 3 and (b) complex 1b and complex 3 for 100 ns. In (a) red color represents complex 1a and cyan color represents complex 3, where in (b) green color represents complex 1b and purple color represents complex 2.

For an in-depth analysis of the interaction between these proteins, the energy contribution of every single residue was calculated and presented in MM/PBSA graphs (Fig. 4). It was observed that, Lys159, Arg230, Lys233, Arg258 and Arg304 from Xa21, and Val2 and Lys15 from RaxX21-sY played an important role during the interaction, by forming MM energy for both complex 1a and complex 3 (Fig. 4a). From Tables S5A and B,† Arg304 of Xa21 shows maximum binding free energy for interacting with RaxX21-sY which is −26.8115 ± 0.1772 kJ mol^−1^ (complex 1a) and −19.3743 ± 0.2091 kJ mol^−1^ (complex 3). Lys159 from Xa21 interacts with RaxX21-sY more favorably in complex 3 (−24.4097 ± 0.1063 kJ mol^−1^), but other residues (Arg230, Lys233, Arg258 and Arg304) show favorable interaction in complex 1a (−20.8768 ± 0.1632 kJ mol^−1^, −21.5039 ± 0.1351 kJ mol^−1^, −21.6513 ± 0.1107 kJ mol^−1^, −26.8115 ± 0.1772 kJ mol^−1^, respectively) (Table S5a†). For RaxX21-sY, Val2 is interacting more favorably with Xa21 in complex 1a (−13.5344 ± 0.0765 kJ mol^−1^) but Lys15 shows favorable interaction in complex 3 (−14.3603 ± 0.5324 kJ mol^−1^) (Table S5C†).

In addition, for the interaction between Xa21 with OsSERK2, several residues contribute significantly to binding energy that are – Arg61, Arg138, Lys159, Arg185, Arg230 and Lys233 for Xa21; and Asp47, Asp80, Lys164, Lys183 and Arg212 for OsSERK2 (Fig. 4b). Lys183 of OsSERK2 shows maximum binding free energy when it interacts with Xa21 which is −50.0972 ± 0.2914 kJ mol^−1^ in complex 1b and −32.3350 ± 0.2338 kJ mol^−1^ in complex 2 (Table S5D†). In both cases, electrostatic interaction is more prominent than van der Waals interaction.

**Fig. 5:**
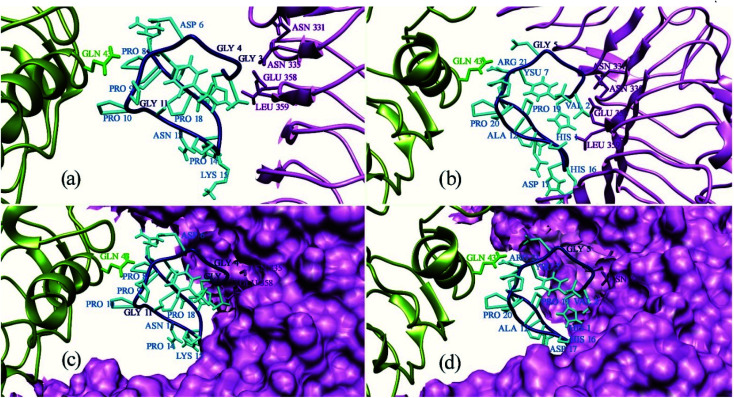
Binding pattern of all residues of PAMP RaxX21-sY (cyan) with Xa21 LRR (purple) and co-receptor OsSERK2 (green). Here, (a) represents binding pattern ten residues of PAMP RaxX21-sY (cyan) with Xa21 LRR (purple) and co-receptor OsSERK2 (green); (b) binding pattern of rest of eleven residues of PAMP RaxX21-sY (cyan) with Xa21 LRR (purple) and co-receptor OsSERK2 (green); (c) binding groove visualization of ten residues of PAMP RaxX21-sY (cyan) with surface structure of Xa21 LRR (purple); (d) binding groove visualization of rest of eleven residues of PAMP RaxX21-sY (cyan) with surface structure of Xa21 LRR (purple).

Protein Interaction Calculator (PIC) online server was employed to analyze the residues involved in interactions both before and after the MD simulation. For the Xa21 and RaxX21-sY interaction, the residues which were found to be the most active from the MM/PBSA calculation, showed participation in different interactions both before and after the simulation process. Most of the residues of RaxX21-sY make strong bonds with Xa21 LRR (Fig. 5a and b) and binds at the inner concave side of the Xa21 LRR to form a sandwich between Xa21 and OsSERK2 (Fig. 5c and d). Val2 forms the protein–protein main chain-side-chain hydrogen bond interaction (Asn331, Phe354, Ala356, Glu358, and Tyr380) as well as hydrophobic interaction with Xa21 (Tyr301) in both complex 1 and complex 3, and Lys15 from RaxX21-sY participates in ionic interactions with Glu358 and Glu407 of Xa21 (Tables S6D and Q†). Similarly, for Xa21, only Arg230 plays a vital role participating in different interactions (mostly ionic interactions) with Asp6 of RaxX21-sY both before and after the simulation (Tables S6B, D and Q†).

Again, for the Xa21 and OsSERK2 interaction (complex 1), Arg185 and Arg230 of Xa21 participate in protein–protein main chain-side chain and side chain-side chain hydrogen bond formation with OsSERK2 residues (Asn50, Leu52, Asp56 and His67) both before and after the simulation (Tables S6A and B†). From OsSERK2 only Lys164 participates in an ionic interaction with Asp565 from Xa21 before and after the simulation. But in complex 2, Lys164 of OsSERk2 participates in a protein–protein main chain-main chain hydrogen bond formation with Thr567 of Xa21 after the simulation (Table S6G†).

### RMS fluctuation (RMSF) of different complexes

**Fig. 6:**
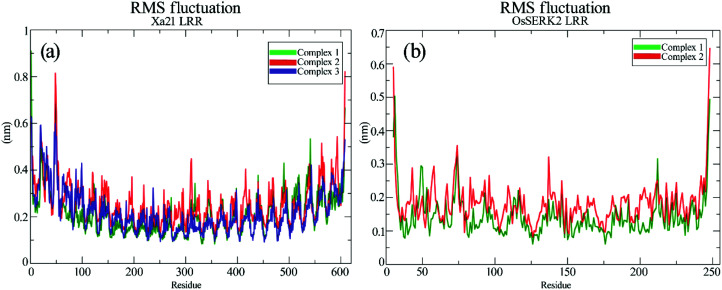
RMS fluctuation of the residues from 100 ns trajectories. (a) RMSF of Xa21 LRR domain, where complex 1 is plotted as green and complex 2 is plotted as red, and complex 3 is plotted as blue; (b) RMSF of co-receptor OsSERK2 LRR domain, where complex 1 is plotted as green and complex 2 is plotted as red.

The RMSFs of the residues of Xa21, RaxX21-sY and OsSERK2 for all the simulated complexes were calculated from the 100 ns MD trajectories. The overall results revealed that most of the residues fluctuated by less than 0.50 nm for Xa21 and RaxX21-sY, and 0.30 nm for OsSERK2. Furthermore, the least amount of fluctuation was displayed by the residues of Xa21 (Lys159, Arg230, Lys233, Arg258 and Arg304) that gave the most favorable MM/PBSA values, in both complex 1a and 3 (Fig. 6a).

Arg230 of Xa21 was found to be the most prominent residue in the MM/PBSA calculation, showed the lowest fluctuation (0.17 nm) in complex 1, as well as the second-lowest fluctuation (0.20 nm) in complex 3. On the other hand, the residues of Xa21 that fluctuated the most are Gln20 (0.57 nm) and Gln541 (0.53) for both complex 1 and complex 3 (Fig. 6a). In the case of RaxX21-sY, Val2 showed the lowest RMSF value of 0.13 nm in complex 1 and a slightly raised value of 0.14 nm in complex 3. Another low fluctuating residue of RaxX21-sY is Lys15 with 0.21 nm in complex 1 and 0.3 nm in complex 3.

When it comes to the Xa21 and OsSERK2 interaction, Arg61, Arg138, Lys159, Arg185, Arg230, Lys233 of Xa21 showed considerably low fluctuations in both complex 1 and complex 2. The MM/PBSA calculation also revealed that these same residues contributed more energy for interacting with OsSERK2. In addition, Arg185 of Xa21 gives the lowest RMSF value both in complex 1 and complex 2 (0.21 nm and 0.27 nm, respectively) (Fig. 6a), which further supports the results of the MM/PBSA calculation. The residues of Xa21 that fluctuated the most in this case were Arg49 and Gln541.

Similarly, for OsSERK2, the residues (Asp47, Asp80, Lys164, Lys183 and Arg212) that had higher energy contribution in the MM/PBSA calculation also gave RMSF values that fluctuated the least in contrast to the other residues of OsSERK2 (Fig. 6b). Among these residues, Lys164 gave the lowest fluctuation value both in complex 1 (0.16 nm) and in complex 2 (0.2 nm) (Fig. 6b).

### Determination of the prominent residues of Xa21 LRR, RaxX21-sY and OsSERK2 LRR

From the overview of MM/PBSA calculations along with an in-depth analysis of the energy contribution of every single residue, PIC data, and RMSF values, some residues can be identified as the major players in the interactions to occur between Xa21, RaxX21-sY and OsSERK2.

For the interaction between Xa21 and RaxX21-sY, Arg230 of Xa21 seems to be this residue as displayed by – the highest MM energy contribution (−51.2735 kJ mol^−1^ and −45.8491 kJ mol^−1^ for complex 1a and complex 3, respectively), the lowest RMS fluctuation, the lowest non-polar energy (Table S5A†), participation in hydrogen bond formation, having hydrophobic interaction and showing electrostatic energy for rising binding affinity with RaxX21-sY (Table S5A†).

When the same analysis of the interactions between Xa21 and OsSERK2 is carried out, Arg185 is found to be the most prominent in every aspect (MM/PBSA, PIC and RMSF calculation). In complex 1b, it gives −113.5355 kJ mol^−1^ MM energy (which primarily comes from electrostatic energy and few contributions from van der Waals energy) and in complex 2, it shows −74.7709 kJ mol^−1^ MM energy which is the highest compared to the other residues of Xa21 (Table S5B†). Moreover, Arg185 of Xa21 also participates in hydrogen bond formation (protein–protein main chain-side chain hydrogen bond, protein–protein side chain-side chain hydrogen bond) with low RMS fluctuation besides the residue Arg230 (Tables S6A and I†).

For RaxX21-sY, though Lys15 contributed more MM energy for interacting with Xa21, Val2 participates in more interactions (protein–protein main chain-side chain hydrogen bond, hydrophobic interaction) with Xa21 (Table S5C†). Therefore, both of these residues can be marked as being prominent residues for the interaction with Xa21.

For OsSERK2, Lys164 showed dominance when interacting with Xa21 with −85.3566 kJ mol^−1^ MM energy which is higher than any other residue of OsSERK2 in complex 1b. In complex 2, the MM energy of Lys164 is −53.8833 kJ mol^−1^ (Table S5D†), which is also high compared to the other residues of OsSERK2. While showing a low RMS fluctuation value, Lys164 also participates in an ionic interaction with Asp565 of Xa21 (Table S6D†) which makes this residue a strong candidate for being a prominent residue.

**Fig. 7:**
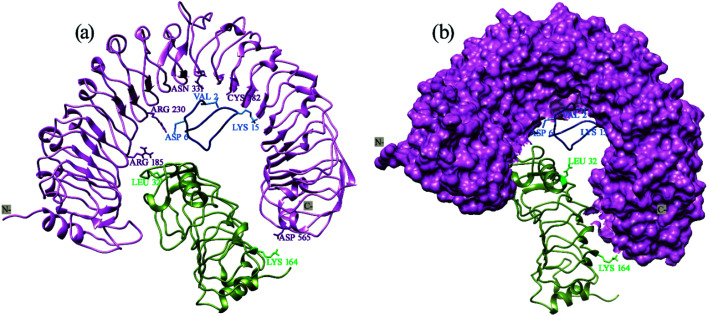
Prominent residues of Xa21 LRR, RaxX21-sY and OsSERK2 LRR. (a) Cartoon and stick structure of the prominent residues of Xa21 LRR (deep purple), RaxX21-sY (cyan) and OsSERK2 (light green); (b) surface structure of the Xa21 LRR (deep purple), and cartoon structure of RaxX21-sY (cyan) and OsSERK2 (light green).

Therefore, from Xa21 Arg230 and Arg185, from RaxX21-sY Val2 and Lys15, and Lys164 of OsSERK2 are the most vital residues when establishing interactions between these three proteins (Fig. 7). Though there are other important residues but in terms of the contribution of van der Waals energy, electrostatic energy, hydrogen bond formation, hydrophobic interactions as well as cation–pi interaction these five residues play the most important role in Xa21 mediated pattern triggered immune complex formation. Thus, it can be hypothesized that a mutation at these residues can greatly affect the plant's ability to trigger pattern triggered immunity.

### Contribution of PAMP RaxX21-sY and co-receptor OsSERK2 in Xa21 mediated PTI

From the MM/PBSA calculation (Table 2), the binding energies of complex 1b and complex 1a are −174.911 kJ mol^−1^ and −45.093 kJ mol^−1^, respectively. Besides these interactions, binding energy of complex 2 is −181.920 kJ mol^−1^, which is more than the value of complex 1b. But an interaction seems unlikely for complex 3 as its binding energy is 10.341 kJ mol^−1^. Moreover, in complex 3, polar solvation energy is high which makes RaxX21-sY non-interactive with Xa21. From this overview, it can be concluded that, co-receptor OsSERK2 plays a significant role for interactions to occur between Xa21 and RaxX21-sY. On the other hand, when it comes to complex 2, the high intermolecular electrostatic energy (Table 2) and MM/PBSA values in contrast to complex 1b suggests that Xa21 can interact with OsSERK2 both in the presence or absence of RaxX21-sY.

**Table 2:** The predicted binding free energy values and the individual energy components for the studied systems (kJ mol^−1^)

Complex	Δ*E*_vdw_^a^	Δ*E*_elec_^b^	Δ*E*_polar_^c^	Δ*E*_sasa_^d^	Δ*E*_bind_^e^
Complex 1a	−240.860 ± 25.595	−385.813 ± 77.663	611.431 ± 91.031	−29.851 ± 2.788	−45.093 ± 52.964
Complex 1b	−286.898 ± 42.162	−606.797 ± 116.533	756.621 ± 157.705	−37.836 ± 7.553	−174.911 ± 110.390
Complex 1c	−96.581 ± 30.324	−85.388 ± 55.586	233.675 ± 97.856	−11.581 ± 3.934	40.124 ± 67.288
Complex 2	−403.270 ± 91.270	−676.877 ± 205.008	946.819 ± 278.196	−48.592 ± 10.988	−181.920 ± 135.765
Complex 3	−250.508 ± 30.032	−362.350 ± 86.456	653.408 ± 119.333	−30.210 ± 2.657	10.341 ± 66.370

a van der Waals energy.

b Electrostatic energy.

c Polar solvation energy.

d Solvent Accessible Surface Area (SASA) energy.

e Binding free energy. Every simulation is performed for 100 ns. Here complex 1a shows interaction between Xa21 and RaxX21-sY when OsSERK2 is present inside the complex, complex 1b shows interaction between Xa21 and OsSERK2 when RaxX21-sY is present and complex 1c for interaction between RaxX21-sY and OsSERK2 when Xa21 is present. Complex 2 indicates the interaction of Xa21 and OsSERK2 in the absence of RaxX21-sY and complex 3 shows interaction of Xa21 and RaxX21-sY in the absence of OsSERK2 inside.

In the case of pattern triggered immunity, most PAMPs recruit one or multiple co-receptors and act as a molecular glue to form a heterodimeric complex of PRR, co-receptor and PAMP. For BRI1, co-receptor SERK1 is being recruited by ligand brassinolide, which initiates the heterodimerization process.^31^ The PSKR-phytosulfokine-SERK1 complex shows the same binding mechanism as BRI1 mediated complex.^13,105^ In addition, in the presence of PAMP IDA, the co-receptor SERK1 binds with HAESA whereas in the absence of IDA the ectodomains of HAESA and SERK1 do not interact with each other.^76^ For FLS2 mediated immunity, flg22 acts as a molecular glue for binding co-receptor BAK1.^14^

Our results show a different pattern of PAMP contribution compared to most of the PTI complexes described above, as the co-receptor OsSERK2 can bind with PRR Xa21 independent of the presence of RaxX21-sY. The previous study on OsSERK2 proved that co-receptor OsSERK2 can frequently bind with PRR Xa21 without the presence of RaxX21-sY^27^ and therefore supports our result. Moreover, our study shows that Xa21 interacts more favorably with OsSERK2 without the presence of RaxX21-sY with binding energy −181.920 kJ mol^−1^.

Though for BRI1, ligand is needed for heterodimerization, it can form homodimer without the presence of ligand brassinolide.^99^ This PAMP's binding to the preformed complexes stabilize the ectodomains of the receptors and activates the kinase domain.^106^ The necessity of these preformed complexes is well defined in the case of receptor tyrosine kinases (RTKs), toll-like receptors (TLRs) and cytokine receptors.^107–109^

Again, one of the key principles of binding for PAMPs is their direction with respect to the PRR's ectodomains. The PAMP and the LRR domain are either oriented in the same direction or in the complete opposite direction.^13,110,111^ In our case, although the structure of RaxX21-sY is slightly different from other PAMPs, it can be said from the docking results that, RaxX21-sY binds inversely with the Xa21 LRR. His1 (N-terminus residue) of RaxX21-sY binds with Asn331, Asn335, Leu359 and Glu358 of Xa21 whereas Arg21 (C-terminus residue) of RaxX21-sY binds with Glu231, Asp253, Gly255 and Tyr279 of Xa21 (Fig. 5).

## Conclusion

This study depicts the in-depth computational modeling of an entire plant PRR-Xa21, and its interaction with PAMP RaxX21-sY and co-receptor OsSERK2 during pattern triggered immunity. Results from different modeling approaches used in this study suggest that a new multiple template approach using the HHpred server described in this paper, is the best for modeling the plant PRR protein Xa21. Likewise, the free energy binding calculation from MM/PBSA uncovers that Arg230 and Arg185 (of Xa21 LRR), Val2, and Lys15 (of RaxX21-sY) and Lys164 (of OsSERK2 LRR) are the key residues which contribute the most in the interaction during PTI. Whether a mutation to these residues affects the rice plants' ability to recognize the PAMP RaxX21-sY is yet to be tested. Another notable finding of this research is the contribution of the co-receptor OsSERK2 when PRR Xa21 interacts with its PAMP RaxX21-sY during PTI. The binding energy between Xa21 LRR and RaxX21-sY is more prominent when OsSERK2 is present in the complex, but in its absence, the energy count is quite low, making interactions between them much less likely. Moreover, this study reveals that Xa21 can interact with co-receptor OsSERK2 even without the presence of PAMP RaxX21-sY, which supports the previous result of heterodimer formation between Xa21 and OsSERK2 in the absence of RaxX21-sY. Though this study exhibits that the interaction between Xa21 and OsSERK2 is more prominent without the presence of RaxX21-sY, a wet-lab experiment needs to be carried out to prove this *in silico* result. As only a crystallographic heterodimer complex of Xa21-RaxX21-sY-OsSERK2 can ascertain the more functional and structural aspects of PTI mediated by Xa21. We strongly believe that all these findings will contribute significantly to the scientific community and enable further study to understand the facets of Xa21 mediated immunity and, overall, the first layer of the plant defense mechanism.

## Funding statement

The author(s) received no financial support for the research, authorship, and/or publication of this article.

## Conflicts of interest

The authors declare that there is no conflict of interest regarding the publication of this paper.

## Supplementary Material

RA-010-D0RA01396J-s001

## References

[cit1] Jones J. D., Dangl J. L. (2006). Nature.

[cit2] Ronald P. C., Beutler B. (2010). Science.

[cit3] Tsuda K., Sato M., Stoddard T., Glazebrook J., Katagiri F. (2009). PLoS Genet..

[cit4] Zipfel C., Robatzek S. (2010). Plant Physiol..

[cit5] Gómez-Gómez L., Boller T. (2000). Mol. Cell.

[cit6] Li J., Chory J. (1997). Cell.

[cit7] Zipfel C., Kunze G., Chinchilla D., Caniard A., Jones J. D., Boller T., Felix G. (2006). Cell.

[cit8] Gust A. A., Felix G. (2014). Curr. Opin. Plant Biol..

[cit9] Liebrand T. W., van den Burg H. A., Joosten M. H. (2014). Trends Plant Sci..

[cit10] Shiu S.-H., Bleecker A. B. (2001). Proc. Natl. Acad. Sci. U. S. A..

[cit11] Fritz-Laylin L. K., Krishnamurthy N., Tör M., Sjölander K. V., Jones J. D. (2005). Plant Physiol..

[cit12] Shiu S.-H., Karlowski W. M., Pan R., Tzeng Y.-H., Mayer K. F., Li W.-H. (2004). Plant Cell.

[cit13] Hohmann U., Lau K., Hothorn M. (2017). Annu. Rev. Plant Biol..

[cit14] Sun Y., Li L., Macho A. P., Han Z., Hu Z., Zipfel C., Zhou J.-M., Chai J. (2013). Science.

[cit15] Dardick C., Ronald P. (2006). PLoS Pathog..

[cit16] Dardick C., Schwessinger B., Ronald P. (2012). Curr. Opin. Plant Biol..

[cit17] Kawai T., Akira S. (2009). Int. Immunol..

[cit18] Wang G.-L., Song W.-Y., Ruan D.-L., Sideris S., Ronald P. C. (1996). Mol. Plant-Microbe Interact..

[cit19] Ishiyama S. , Report of the Imperial Agricultural Station, Nishigahara, Konosu, 1922, vol. 45, pp. 233–261

[cit20] Swings J., Van den Mooter M., Vauterin L., Hoste B., Gillis M., Mew T., Kersters K. (1990). Int. J. Syst. Evol. Microbiol..

[cit21] Mew T. (1987). Annu. Rev. Phytopathol..

[cit22] Niño-Liu D. O., Ronald P. C., Bogdanove A. J. (2006). Mol. Plant Pathol..

[cit23] Pinta W., Toojinda T., Thummabenjapone P., Sanitchon J. (2013). Afr. J. Biotechnol..

[cit24] Song W.-Y., Wang G.-L., Chen L.-L., Kim H.-S., Pi L.-Y., Holsten T., Gardner J., Wang B., Zhai W.-X., Zhu L.-H. (1995). Science.

[cit25] Pruitt R. N., Schwessinger B., Joe A., Thomas N., Liu F., Albert M., Robinson M. R., Chan L. J. G., Luu D. D., Chen H. (2015). Sci. Adv..

[cit26] Mubassir M., Naser M. A., Abdul-Wahab M. F., Hamdan S. (2019). J. Chem. Pharm. Sci..

[cit27] Chen X., Zuo S., Schwessinger B., Chern M., Canlas P. E., Ruan D., Zhou X., Wang J., Daudi A., Petzold C. J. (2014). Mol. Plant.

[cit28] Schwessinger B., Li X., Ellinghaus T. L., Chan L. J. G., Wei T., Joe A., Thomas N., Pruitt R., Adams P. D., Chern M. S. (2016). Integr. Biol..

[cit29] Wei T., Chern M., Liu F., Ronald P. C. (2016). Mol. Plant Pathol..

[cit30] Pruitt R. N., Joe A., Zhang W., Feng W., Stewart V., Schwessinger B., Dinneny J. R., Ronald P. C. (2017). New Phytol..

[cit31] Hothorn M., Belkhadir Y., Dreux M., Dabi T., Noel J. P., Wilson I. A., Chory J. (2011). Nature.

[cit32] Jinn T.-L., Stone J. M., Walker J. C. (2000). Genes Dev..

[cit33] Sindhu T., Srinivasan P. (2015). Mol. BioSyst..

[cit34] Gasteiger E. , Hoogland C., Gattiker A., Wilkins M. R., Appel R. D. and Bairoch A., in The proteomics protocols handbook, Springer, 2005, pp. 571–607

[cit35] McGuffin L. J., Bryson K., Jones D. T. (2000). Bioinformatics.

[cit36] Geourjon C., Deleage G. (1995). Bioinformatics.

[cit37] Armon A., Graur D., Ben-Tal N. (2001). J. Mol. Biol..

[cit38] Hunter S., Apweiler R., Attwood T. K., Bairoch A., Bateman A., Binns D., Bork P., Das U., Daugherty L., Duquenne L. (2008). Nucleic Acids Res..

[cit39] Schultz J., Milpetz F., Bork P., Ponting C. P. (1998). Proc. Natl. Acad. Sci. U. S. A..

[cit40] Offord V., Coffey T., Werling D. (2010). Dev. Comp. Immunol..

[cit41] Biegert A., Söding J. (2008). Bioinformatics.

[cit42] Rost B., Yachdav G., Liu J. (2004). Nucleic Acids Res..

[cit43] Emanuelsson O., Brunak S., Von Heijne G., Nielsen H. (2007). Nat. Protoc..

[cit44] Nugent T., Jones D. T. (2009). BMC Bioinf..

[cit45] Hirokawa T., Boon-Chieng S., Mitaku S. (1998). Bioinformatics.

[cit46] Mahram A. and Herbordt M. C., Proceedings of the 24th ACM International Conference on Supercomputing, 2010, pp. 73–82

[cit47] Söding J., Biegert A., Lupas A. N. (2005). Nucleic Acids Res..

[cit48] Eswar N., Webb B., Marti-Renom M. A., Madhusudhan M., Eramian D., Shen M. y., Pieper U., Sali A. (2006). Curr. Protoc. Bioinf..

[cit49] Bates P. A., Kelley L. A., MacCallum R. M., Sternberg M. J. (2001). Proteins: Struct., Funct., Bioinf..

[cit50] Nielsen M., Lundegaard C., Lund O., Petersen T. N. (2010). Nucleic Acids Res..

[cit51] Combet C., Jambon M., Deleage G., Geourjon C. (2002). Bioinformatics.

[cit52] Yang Y., Faraggi E., Zhao H., Zhou Y. (2011). Bioinformatics.

[cit53] Chen C.-C., Hwang J.-K., Yang J.-M. (2006). Nucleic Acids Res..

[cit54] Lobley A., Sadowski M. I., Jones D. T. (2009). Bioinformatics.

[cit55] Xu D., Jaroszewski L., Li Z., Godzik A. (2013). Bioinformatics.

[cit56] Jaroszewski L., Rychlewski L., Li Z., Li W., Godzik A. (2005). Nucleic Acids Res..

[cit57] Zhou H., Skolnick J. (2009). Biophys. J..

[cit58] Huang Y. J., Mao B., Aramini J. M., Montelione G. T. (2014). Proteins: Struct., Funct., Bioinf..

[cit59] Schwede T., Kopp J., Guex N., Peitsch M. C. (2003). Nucleic Acids Res..

[cit60] Wu S., Zhang Y. (2008). Proteins: Struct., Funct., Bioinf..

[cit61] Yang J., Yan R., Roy A., Xu D., Poisson J., Zhang Y. (2015). Nat. Methods.

[cit62] Simossis V. A., Heringa J. (2005). Nucleic Acids Res..

[cit63] Kelley L. A., Mezulis S., Yates C. M., Wass M. N., Sternberg M. J. (2015). Nat. Protoc..

[cit64] Källberg M. , Margaryan G., Wang S., Ma J. and Xu J., in Protein Structure Prediction, Springer, 2014, pp. 17–27 10.1007/978-1-4939-0366-5_2 24573471

[cit65] Zhang Y. (2008). BMC Bioinf..

[cit66] Xu D., Jaroszewski L., Li Z., Godzik A. (2014). Nucleic Acids Res..

[cit67] Colovos C., Yeates T. (1993). Protein Sci..

[cit68] Eisenberg D. , Lüthy R. and Bowie J. U., in Methods in enzymology, Elsevier, 1997, vol. 277, pp. 396–404 10.1016/s0076-6879(97)77022-8 9379925

[cit69] Laskowski R. A., MacArthur M. W., Moss D. S., Thornton J. M. (1993). J. Appl. Crystallogr..

[cit70] DeLano W. L. (2002). J. Med. Chem..

[cit71] Wallner B., Fang H., Elofsson A. (2003). Proteins: Struct., Funct., Bioinf..

[cit72] Wiederstein M., Sippl M. J. (2007). Nucleic Acids Res..

[cit73] Willard L., Ranjan A., Zhang H., Monzavi H., Boyko R. F., Sykes B. D., Wishart D. S. (2003). Nucleic Acids Res..

[cit74] Chen V. B., Arendall W. B., Headd J. J., Keedy D. A., Immormino R. M., Kapral G. J., Murray L. W., Richardson J. S., Richardson D. C. (2010). Acta Crystallogr., Sect. D: Biol. Crystallogr..

[cit75] Wang G.-L., Ruan D.-L., Song W.-Y., Sideris S., Chen L., Pi L.-Y., Zhang S., Zhang Z., Fauquet C., Gaut B. S. (1998). Plant Cell.

[cit76] Santiago J., Brandt B., Wildhagen M., Hohmann U., Hothorn L. A., Butenko M. A., Hothorn M. (2016). eLife.

[cit77] She J., Han Z., Kim T.-W., Wang J., Cheng W., Chang J., Shi S., Wang J., Yang M., Wang Z.-Y. (2011). Nature.

[cit78] Zhang Z., Thomma B. P. (2013). J. Integr. Plant Biol..

[cit79] Andaya C. B., Ronald P. C. (2003). Physiol. Mol. Plant Pathol..

[cit80] Pierce B. G., Wiehe K., Hwang H., Kim B.-H., Vreven T., Weng Z. (2014). Bioinformatics.

[cit81] Van Der Spoel D., Lindahl E., Hess B., Groenhof G., Mark A. E., Berendsen H. J. (2005). J. Comput. Chem..

[cit82] Schmid N., Eichenberger A. P., Choutko A., Riniker S., Winger M., Mark A. E., van Gunsteren W. F. (2011). Eur. Biophys. J..

[cit83] van der Spoel D., van Maaren P. J., Berendsen H. J. (1998). J. Chem. Phys..

[cit84] Pettersen E. F., Goddard T. D., Huang C. C., Couch G. S., Greenblatt D. M., Meng E. C., Ferrin T. E. (2004). J. Comput. Chem..

[cit85] Genheden S., Ryde U. (2015). Expert Opin. Drug Discovery.

[cit86] Kumari R., Kumar R., Consortium O. S. D. D., Lynn A. (2014). J. Chem. Inf. Model..

[cit87] DeLano W. L. , CCP4 Newsletter on protein crystallography, 2002, vol. 40, pp. 82–92

[cit88] Wang G.-L., Song W.-Y., Ruan D.-L., Sideris S., Ronald P. C. (1996). Mol. Plant-Microbe Interact..

[cit89] Buenavista M. T., Roche D. B., McGuffin L. J. (2012). Bioinformatics.

[cit90] Kobe B., Kajava A. V. (2001). Curr. Opin. Struct. Biol..

[cit91] Wang J., Li H., Han Z., Zhang H., Wang T., Lin G., Chang J., Yang W., Chai J. (2015). Nature.

[cit92] Song W., Han Z., Sun Y., Chai J. (2014). Sci. China: Life Sci..

[cit93] Wang J., Li H., Han Z., Zhang H., Wang T., Lin G., Chang J., Yang W., Chai J. (2015). Nature.

[cit94] Albert M., Felix G. (2010). Plant Signaling Behav..

[cit95] Bojar D., Martinez J., Santiago J., Rybin V., Bayliss R., Hothorn M. (2014). Plant J..

[cit96] Yan L., Ma Y., Liu D., Wei X., Sun Y., Chen X., Zhao H., Zhou J., Wang Z., Shui W. (2012). Cell Res..

[cit97] Oh M.-H., Clouse S. D., Huber S. C. (2012). Front. Plant Sci..

[cit98] Wang X., Kota U., He K., Blackburn K., Li J., Goshe M. B., Huber S. C., Clouse S. D. (2008). Dev. Cell.

[cit99] Wang X., Li X., Meisenhelder J., Hunter T., Yoshida S., Asami T., Chory J. (2005). Dev. Cell.

[cit100] Xu W. H., Wang Y. S., Liu G. Z., Chen X., Tinjuangjun P., Pi L. Y., Song W. Y. (2006). Plant J..

[cit101] Park C. J., Han S. W., Chen X., Ronald P. C. (2010). Cell. Microbiol..

[cit102] Wang Y., Pi L., Chen X., Chakrabarty P., Jiang J. (2006). Plant Cell.

[cit103] Chen X., Chern M., Canlas P. E., Jiang C., Ruan D., Cao P., Ronald P. C. (2010). J. Biol. Chem..

[cit104] Tang J., Han Z., Sun Y., Zhang H., Gong X., Chai J. (2015). Cell Res..

[cit105] Hartmann J., Fischer C., Dietrich P., Sauter M. (2014). Plant J..

[cit106] Albrecht C., Boutrot F., Segonzac C., Schwessinger B., Gimenez-Ibanez S., Chinchilla D., Rathjen J. P., de Vries S. C., Zipfel C. (2012). Proc. Natl. Acad. Sci. U. S. A..

[cit107] Lemmon M. A., Schlessinger J. (2010). Cell.

[cit108] Kang J. Y., Lee J.-O. (2011). Annu. Rev. Biochem..

[cit109] Wang X., Lupardus P., LaPorte S. L., Garcia K. C. (2009). Annu. Rev. Immunol..

[cit110] Shinohara H., Mori A., Yasue N., Sumida K., Matsubayashi Y. (2016). Proc. Natl. Acad. Sci. U. S. A..

[cit111] Somssich M., Ma Q., Weidtkamp-Peters S., Stahl Y., Felekyan S., Bleckmann A., Seidel C. A., Simon R. (2015). Sci. Signaling.

