# Supplementary material for: Comprehensive in silico modeling of the rice plant PRR Xa21 and its interaction with RaxX21-sY and OsSERK2
Source: RSC Adv. 2020 Apr 21;10(27):15800–14. doi: 10.1039/d0ra01396j (PMC9052883; doi:10.1039/d0ra01396j)
Supplement: RA-010-D0RA01396J-s001 [file RA-010-D0RA01396J-s001.pdf]

Electronic Supplementary Material (ESI) for RSC Advances.
This journal is © The Royal Society of Chemistry 2020

ELECTRONIC SUPPLEMENTARY INFORMATION (ESI)

Comprehensive *in silico* modeling of plant PRR Xa21 and its interaction with RaxX21-sY and OsSERK2

M H M Mubassir,*[a] M Abu Naser,[b] Mohd Firdaus Abdul-Wahab,[b] Tanvir Jawad,[a] Raghib Ishraq Alvy[a] and Salehhuddin Hamdan *[b]

[a.] *Department of Mathematics and Natural Sciences, BRAC University, 66 Mohakhali, Dhaka-1212, Bangladesh.*
[b.] *Faculty Bioscience and Medical Engineering, Universiti Teknologi Malaysia, 81310 Johor Bahru, Johor, Malaysia*

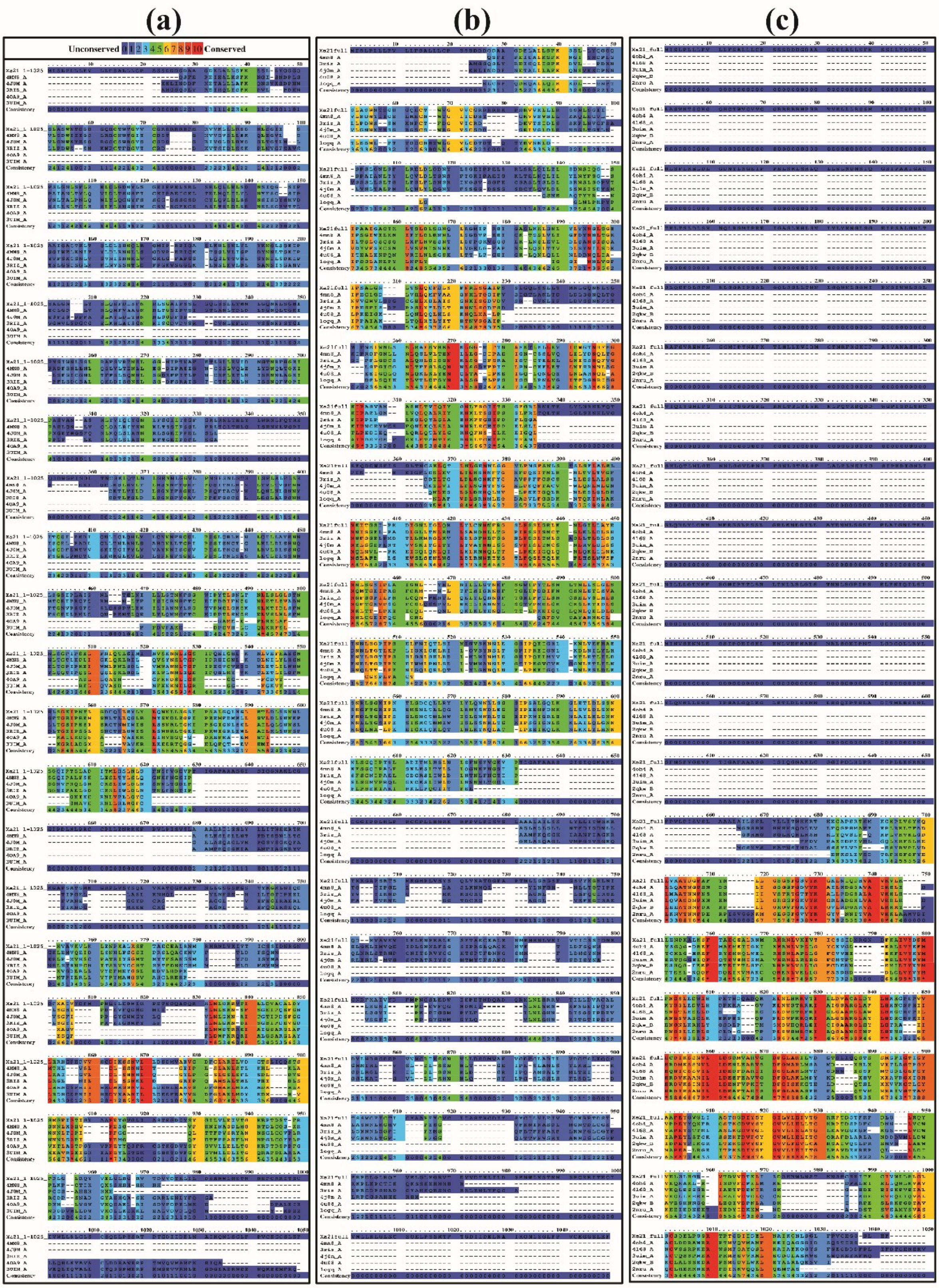

Fig. S1 Multiple sequence alignment (MSA) of Xa21 protein sequence and top five BLASTP results for (a) Full sequence 1-1025 (b) LRR templates (c) JM and Kinase templates

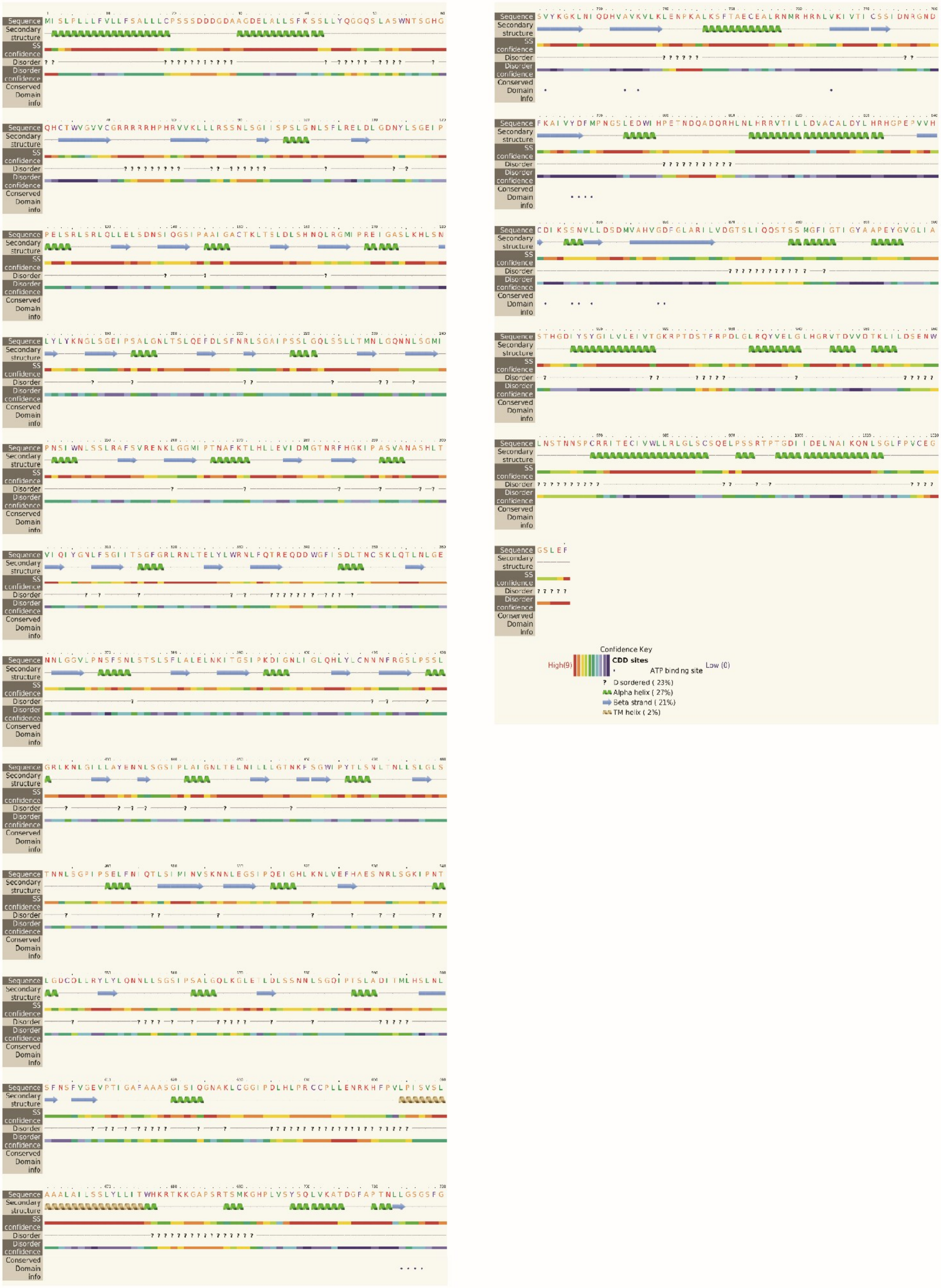

Fig. S2 Secondary structure of Xa21 protein predicted by PSIPRED

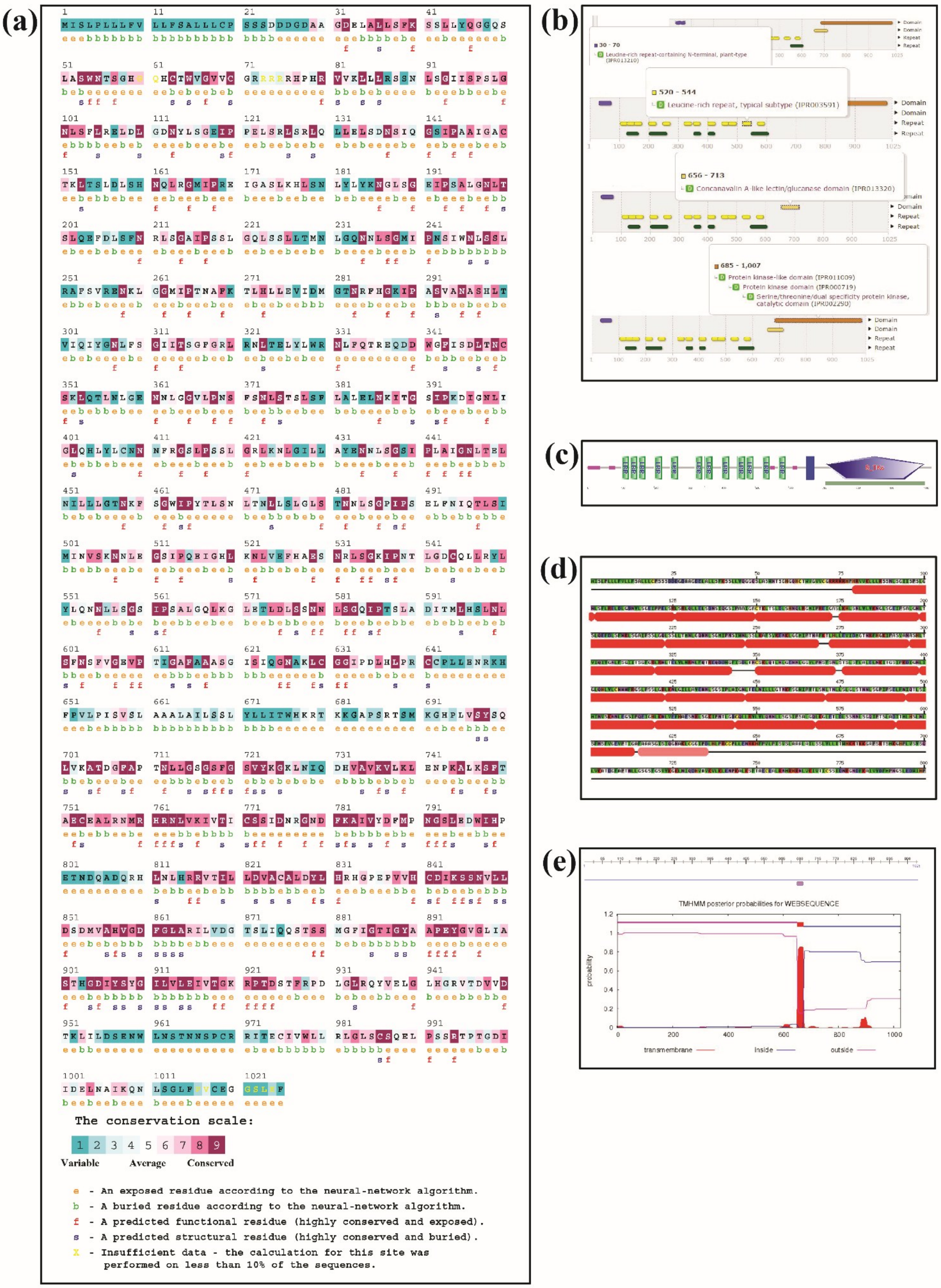

Fig. S3 (a) Conserved region prediction of Xa21 by the ConSurf tool. (b) Domain boundary prediction of Xa21 using InterPro. (c) Domain boundary prediction of Xa21 by the SMART tool. (d) Repeated Xa21 LRR region prediction by the HHrepID tool. (e) Transmembrane region prediction of Xa21 protein by the TMHMM tool.

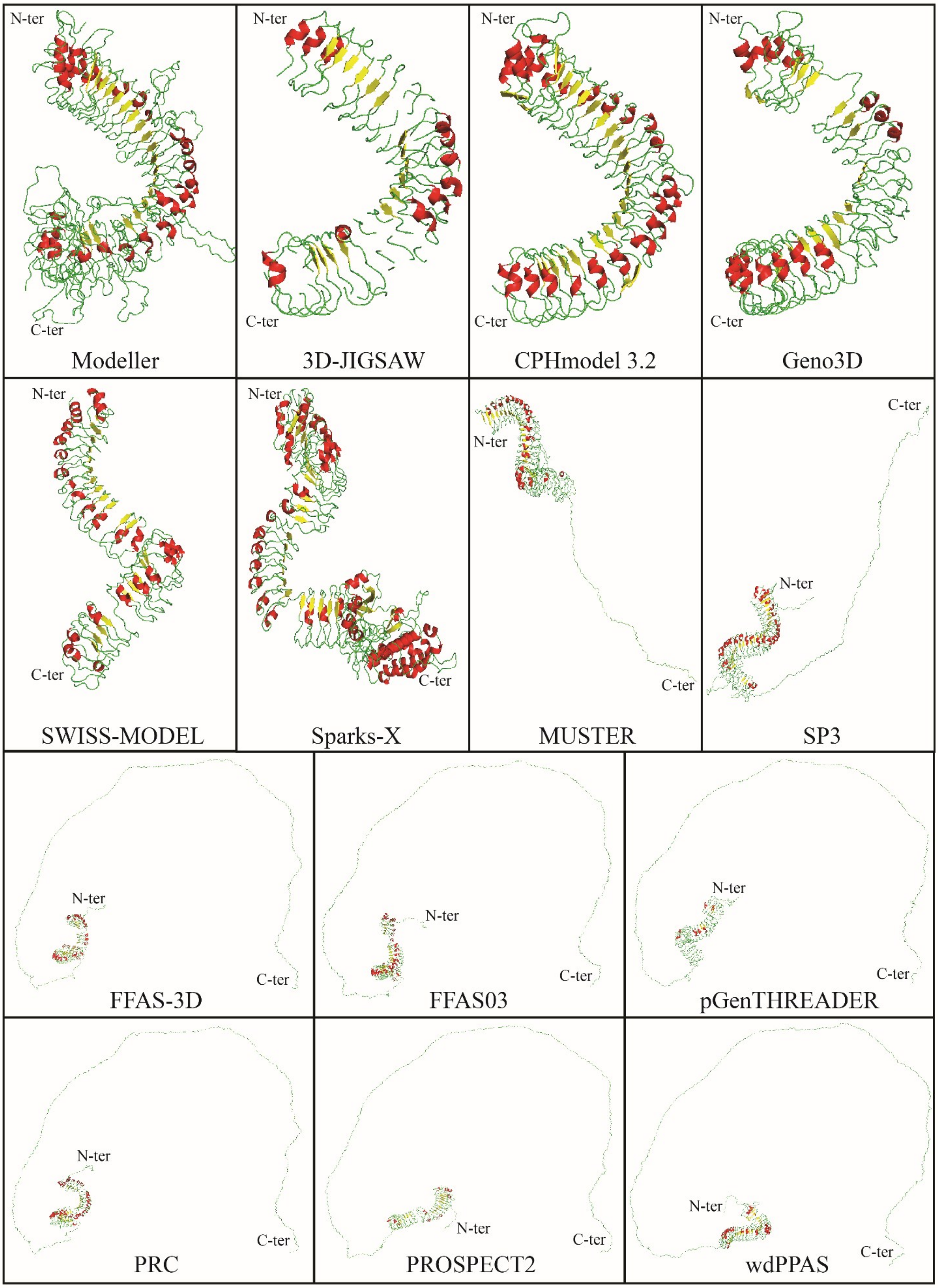

Fig. S4 Cartoon representations of the Xa21 proteins modeled using single template modeling approaches. Red colored regions indicate alpha helixes, yellow colored regions indicate beta sheets and green colored regions indicate coiled regions.

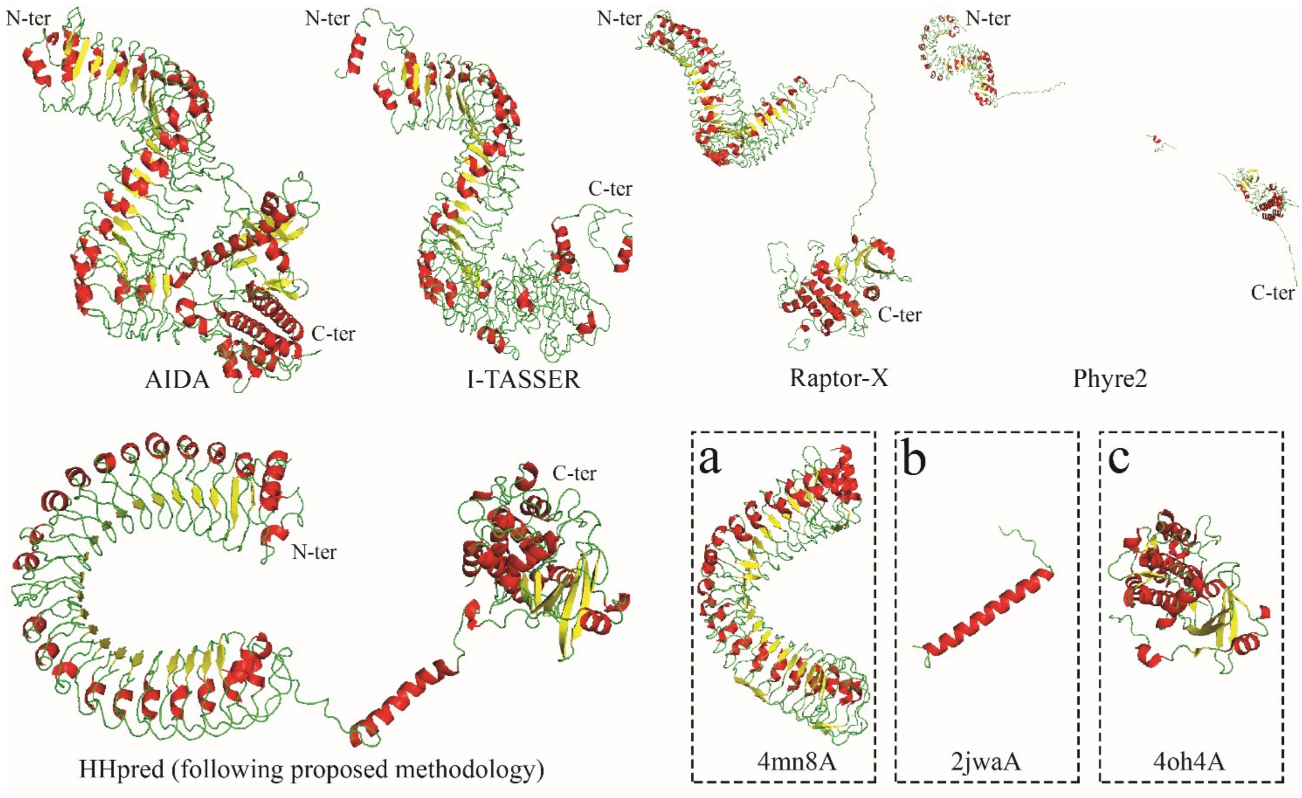

Fig. S5. Cartoon representations of the Xa21 protein modeled using multiple template modeling approaches. The boxed proteins are 3D structures of the best template from the PDB - (a) LRR domain, (b) TM domain, (c) Kinase domain, of Xa21. Red colored regions indicate alpha helixes, yellow colored regions indicate beta sheets and green colored regions indicate coil regions.

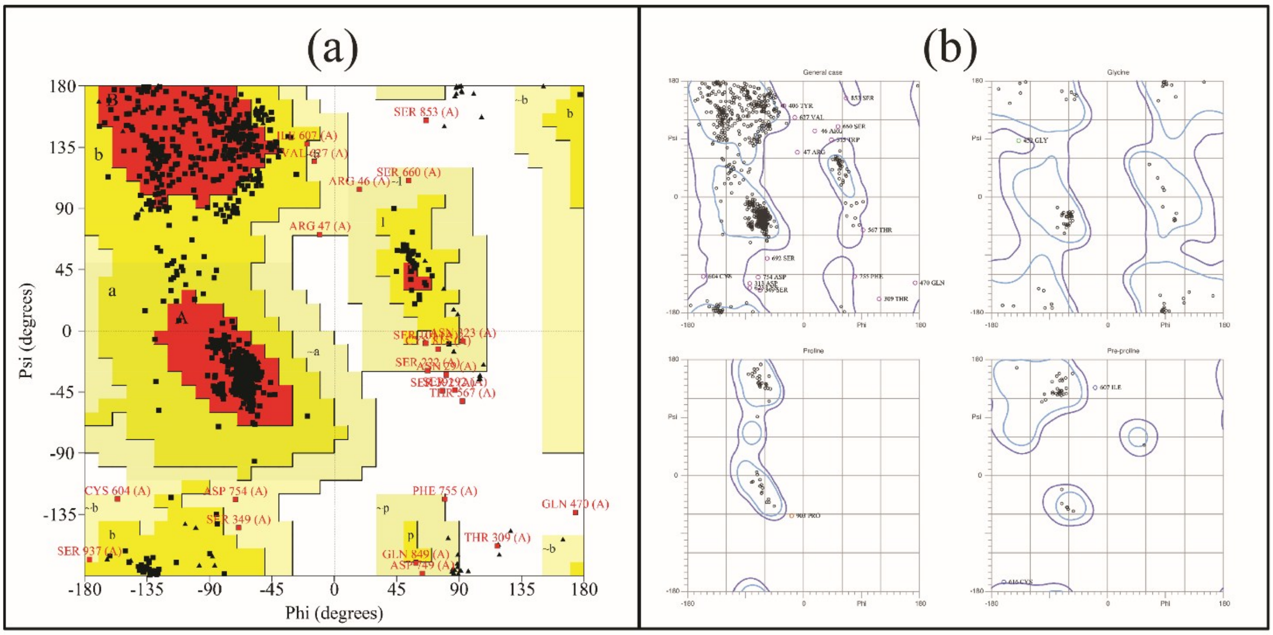

Fig. S6. Ramachandran plot summery for Xa21 protein modeled by the HHpred server is on the right-hand side and the left-hand side figure indicates the MolProbity for the Ramachandran plot.

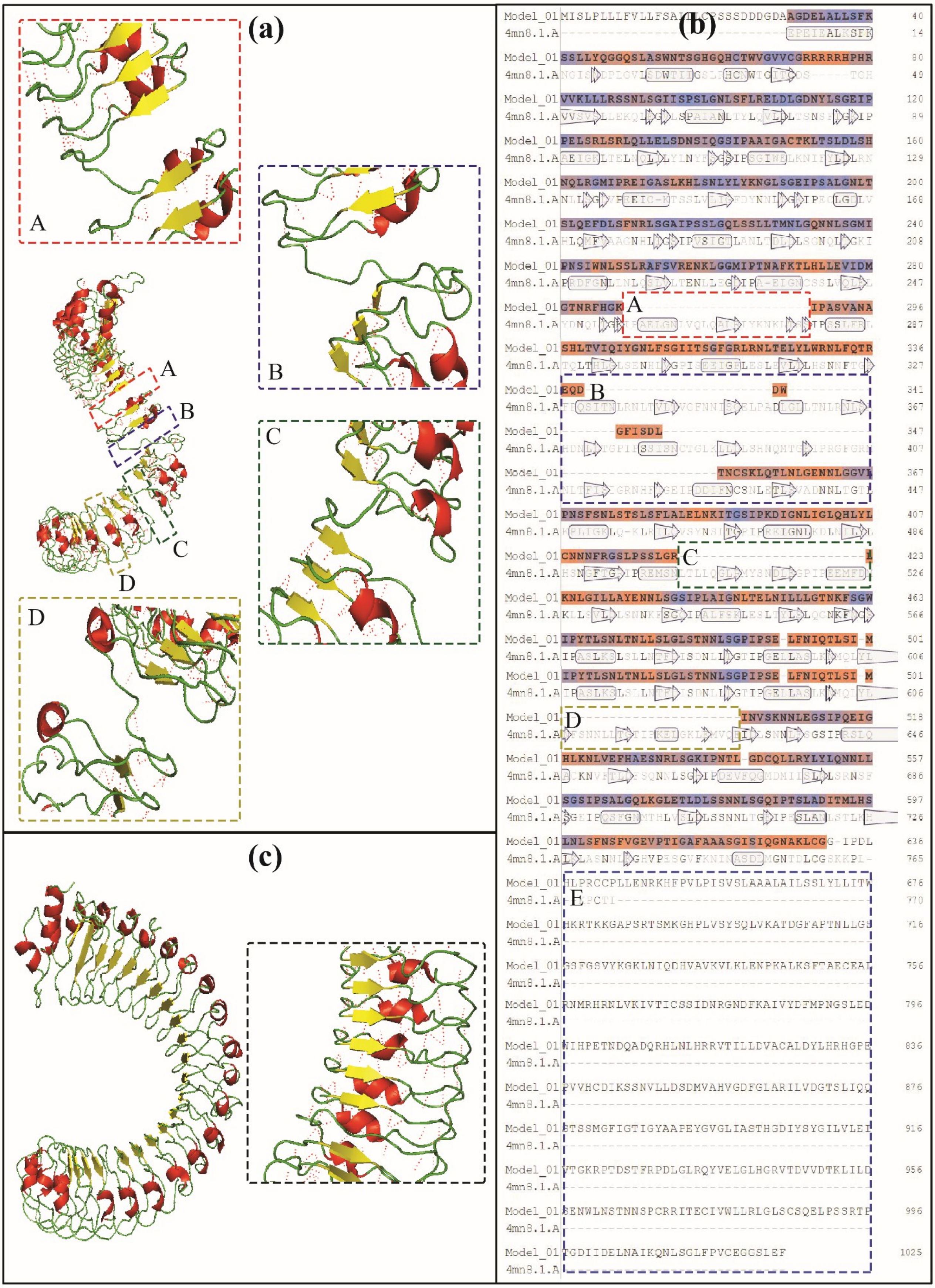

Fig. S7. (a) Cartoon representation of the Xa21 protein modeled by the Swiss-model tool showing the different gaps in the structure. (b) Alignment of the modeled protein sequence and the template sequence by Swiss-model. (c) Cartoon representation of LRR domain modeled by the HHpred server. Red colored regions indicate alpha helix, yellow colored regions indicate beta sheet and green colored regions indicate coil region.

Table S1A. Modeling methodology of the Xa21 Protein using Single Template Modeling approach

Tool	Modeling Method	Template
Modeller 9.15	Homology	4mn8A
3D-JIGSAW	Homology	4mn8A
CPHmodel 3.2	Homology	4mn8A
Geno3D	Homology	4mn8A
Swissmodel	Homology	4mn8A
PRC	Threading	4mn8A
pGenTHREADER	Threading	4mn8A
Prospect2	Threading	4mn8A
FFAS-3D	Threading	4mn8A
FFAS03	Threading	4mn8A
SP3	Threading	4mn8A
Sparks-X	Threading	4mn8A
Musterm	Threading	4mn8A
WdPPAS	Threading	4j0mA

Table S1B. Modeling methodology of the Xa21 Protein using Multiple Template Modeling Approach

R/P ID	R/P Name	AA boundary	Tool	Modeling Method	Template(s)
Xa21lrr_1	N-term+LRR	27-634	HHpred	Homology	4mn8A
Xa21lrr_2	N-term+LRR	27-634	HHpred	Homology	4mn8A+4j0mA
Xa21lrr_3	N-term+LRR	27-634	HHpred	Homology	4mn8A+3rgzA
Xa21lrr_4	N-term+LRR	27-634	HHpred	Homology	4mn8A+1ogqA
Xa21lrr_5	N-term+LRR	27-634	HHpred	Homology	4mn8A+4j0mA+1ogqA
Xa21lrr_6	N-term+LRR	27-634	HHpred	Homology	4mn8A+1ogqA+3rgzA
Xa21lrr_7	N-term+LRR	27-634	HHpred	Homology	4mn8A+3rgzA+4j0mA
Xa21lrr_8	N-term+LRR	27-634	HHpred	Homology	4mn8A+1ogqA+4j0mA+3rgzA
Xa21lrr_9	N-term+LRR	27-634	HHpred	Homology	4mn8A+4u08A
Xa21lrr_10	N-term+LRR	27-634	HHpred	Homology	4mn8A+4u08A+3rgzA
Xa21lrr_11	N-term+LRR	27-634	HHpred	Homology	4mn8A+4u08A+4j0mA
Xa21lrr_12	N-term+LRR	27-634	HHpred	Homology	4mn8A+4u08A+1ogqA
Xa21lrr_13	N-term+LRR	27-634	HHpred	Homology	4mn8A+4u08A+3rgzA+4j0mA
Xa21lrr_14	N-term+LRR	27-634	HHpred	Homology	4mn8A+4u08A+4j0mA+1ogqA
Xa21lrr_15	N-term+LRR	27-634	HHpred	Homology	4mn8A+4u08A+4j0mA+3rgzA+1ogqA
Xa21lrr_16	N-term+LRR	27-634	HHpred	Homology	4u08A+3rgzA
Xa21lrr_17	N-term+LRR	27-634	HHpred	Homology	4u08A+4j0mA
Xa21lrr_18	N-term+LRR	27-634	HHpred	Homology	4u08A+1ogqA
Xa21lrr_19	N-term+LRR	27-634	HHpred	Homology	3rgzA+4j0mA
Xa21lrr_20	N-term+LRR	27-634	HHpred	Homology	3rgzA+1ogqA
Xa21lrr_21	N-term+LRR	27-634	HHpred	Homology	4j0mA+1ogqA
Xa21lrr_22	N-term+LRR	27-634	HHpred	Homology	4u08A+3rgzA+4j0mA
Xa21lrr_23	N-term+LRR	27-634	HHpred	Homology	4u08A+3rgzA+1ogqA
Xa21lrr_24	N-term+LRR	27-634	HHpred	Homology	4u08A+4j0mA+1ogqA
Xa21lrr_25	N-term+LRR	27-634	HHpred	Homology	3rgzA+4j0mA+1ogqA

Xa21lrr_26	N-term+LRR	27-634	HHpred	Homology	4u08A+3rgzA+4j0mA+1ogqA
Xa21lrr_27	N-term+LRR	27-634	HHpred	Homology	3rgzA
Xa21lrr_28	N-term+LRR	27-634	HHpred	Homology	4j0mA
Xa21lrr_29	N-term+LRR	27-634	HHpred	Homology	1ogqA
Xa21lrr_30	N-term+LRR	27-634	HHpred	Homology	4u08A
Xa21lrr_31	N-term+LRR	27-634	HHpred	Homology	4mn8A+4u08A+3rgzA+1ogqA
Xa21c12tm_1	TM+c1+c2	651-707	HHpred	Homology	2jwaA
Xa21c12tm_2	TM+c1+c2	651-707	HHpred	Homology	2ks1B
Xa21c12tm_3	TM+c1+c2	651-707	HHpred	Homology	2jwaA+2ks1B
Xa21k_1	JM+K+C-term	708-1004	HHpred	Homology	4oh4A
Xa21k_2	JM+K+C-term	708-1004	HHpred	Homology	3uimA
Xa21k_3	JM+K+C-term	708-1004	HHpred	Homology	4l68A
Xa21k_4	JM+K+C-term	708-1004	HHpred	Homology	2nruA
Xa21k_5	JM+K+C-term	708-1004	HHpred	Homology	2qkwB
Xa21k_6	JM+K+C-term	708-1004	HHpred	Homology	4oh4A+3uimA
Xa21k_7	JM+K+C-term	708-1004	HHpred	Homology	4oh4A+4l68A
Xa21k_8	JM+K+C-term	708-1004	HHpred	Homology	4oh4A+2nruA
Xa21k_9	JM+K+C-term	708-1004	HHpred	Homology	4oh4A+2qkwB
Xa21k_10	JM+K+C-term	708-1004	HHpred	Homology	3uimA+4l68A
Xa21k_11	JM+K+C-term	708-1004	HHpred	Homology	3uimA+2nruA
Xa21k_12	JM+K+C-term	708-1004	HHpred	Homology	3uimA+2qkwB
Xa21k_13	JM+K+C-term	708-1004	HHpred	Homology	4l68A+2nruA
Xa21k_14	JM+K+C-term	708-1004	HHpred	Homology	4l68A+2qkwB
Xa21k_15	JM+K+C-term	708-1004	HHpred	Homology	2nruA+2qkwB
Xa21k_16	JM+K+C-term	708-1004	HHpred	Homology	40h4A+3uimA+4l68A
Xa21k_17	JM+K+C-term	708-1004	HHpred	Homology	4oh4A+3uimA+2nruA
Xa21k_18	JM+K+C-term	708-1004	HHpred	Homology	4oh4A+3uimA+2qkwB
Xa21k_19	JM+K+C-	708-1004	HHpred	Homology	3uimA+4l68A+2nruA

	term				
Xa21k_20	JM+K+C-term	708-1004	HHpred	Homology	3uimA+4l68A+2qkwB
Xa21k_21	JM+K+C-term	708-1004	HHpred	Homology	4l68A+2nruA+2qkwB
Xa21k_22	JM+K+C-term	708-1004	HHpred	Homology	40h4A+2nruA+2qkwB
Xa21k_23	JM+K+C-term	708-1004	HHpred	Homology	3uimA+4l68A+2qkwB
Xa21k_24	JM+K+C-term	708-1004	HHpred	Homology	3uimA+2nruA+2qkwB
Xa21k_25	JM+K+C-term	708-1004	HHpred	Homology	4oh4A+4l68A+2qkwB
Xa21k_26	JM+K+C-term	708-1004	HHpred	Homology	4oh4A+4l68A+2nruA+2qkwB
Xa21k_27	JM+K+C-term	708-1004	HHpred	Homology	4oh4A+3uimA+4l68A+2nruA
Xa21k_28	JM+K+C-term	708-1004	HHpred	Homology	3uimA+4l68A+2nruA+2qkwB
Xa21k_29	JM+K+C-term	708-1004	HHpred	Homology	4oh4A+4l68A+2nruA+2qkwB
Xa21k_30	JM+K+C-term	708-1004	HHpred	Homology	4oh4A+3uimA+4l68A+2nruA+2qkwB
Xa21k_31	JM+K+C-term	708-1004	HHpred	Homology	4oh4A+3uimA+2nruA+2qkwB
Xa21_Final	Xa21	27-1009	HHpred	Homology	Xa21lrr_15+Xa21c12tm_3+Xa21k_1
Xa21_AIDA	Xa21	27-1009	AIDA	Homology	4mnA+2jwA+4l68A
Xa21_I-Tas	Xa21	1-1025	I-Tasser	*Ab initio*& Threading	4mn8A(7)+4oa2A+4j0mA
Xa21_PhyI	Xa21	1-1025	Phyre2 Intensive	Homology&*Ab initio*	4mn8A(2)+4y93A+2j0kB+1oplA+4xi2A+1y57A+2fo0A
Xa21_RapX	Xa21	1-1025	Raptor-X	Threading	4mn8A+3tl8A+4oa2A+4oa6A

R/P ID, Region or Protein ID; R/P Name, Region or protein Name; AA, Amino Acid; LRR, Leucine rich repeat; TM+C1+C2, Transmembrane and Charged 1 and Charged 2 region; JM+K+C-term, Juxtamembrane and Kinase and C terminal region.

Table S2A. NCBI BLASP analysis of different domain regions of Xa21

Region	AA Boundary	PDB ID	Max. Score	E-value	Q C (%)	Idn. (%)	Template Short Identity
**Xa21 full**	**1-1025**	4mn8_A	311	4.00E-91	58	33	Chain A, Crystal Structure Of Flg22 In Complex With The Fls2 And Bak1 Ectodomains
		4j0m_A	206	3.00E-55	54	33	Chain A, Crystal Structure Of Brl1 (lrr) In Complex With Brassinolide
		3riz_A	201	2.00E-53	54	33	Chain A, Crystal Structure Of The Plant Steroid Receptor Bri1 Ectodomain
		4oa9_A	169	1.00E-45	25	38	Chain A, Crystal Structure Of The Bri1 Kinase Domain (865-1160) In Complex With Amppnp And Mn From Arabidopsis Thaliana
		3uim_A	129	8.00E-32	22	36	Chain A, Structural Basis For The Impact Of Phosphorylation On Plant Receptor- Like Kinase Bak1 Activation
**Xa21**	**27-1009**	4mn8_A	312	1.00E-91	61	33	Chain A, Crystal Structure Of Flg22 In Complex With The Fls2 And Bak1 Ectodomains
		4j0m_A	206	1.00E-55	57	33	Chain A, Crystal Structure Of Brl1 (lrr) In Complex With Brassinolide
		3riz_A	201	9.00E-54	57	33	Chain A, Crystal Structure Of The Plant Steroid Receptor Bri1 Ectodomain
		4oa9_A	169	8.00E-46	26	38	Chain A, Crystal Structure Of The Bri1 Kinase Domain (865-1160) In Complex With Amppnp And Mn From Arabidopsis Thaliana
		3uim_A	129	7.00E-32	23	36	Chain A, Structural Basis For The Impact Of Phosphorylation On Plant Receptor- Like Kinase Bak1 Activation
**N-term+LRR**	**27-634**	4mn8_A	312	4.00E-95	98	33	Chain A, Crystal Structure Of Flg22 In Complex With The Fls2 And Bak1 Ectodomains
		3riz_A	212	7.00E-59	91	32	Chain A, Crystal Structure Of The

							Plant Steroid Receptor Bri1 Ectodomain
		4j0m_A	207	3.00E-57	92	33	Chain A, Crystal Structure Of Brl1 (lrr) In Complex With Brassinolide
		1ogq_A	105	2.00E-24	98	32	Chain A, The Crystal Structure Of Pgip (Polygalacturonase Inhibiting Protein), A Leucine Rich Repeat Protein Involved In Plant Defense
		4u08_A	103	2.00E-23	67	29	Chain A, Structure Of LeptospiraInterrogansLrr Protein Lic11098
**C1+TM+C2+JM+K**	**635-1009**	4oh4_A	166	7.00E-47	69	38	Chain A, Crystal Structure Of Bri1 In Complex With Bki1
		3uim_A	130	7.00E-34	60	36	Chain A, Structural Basis For The Impact Of Phosphorylation On Plant Receptor- Like Kinase Bak1 Activation
		4l68_A	108	2.00E-26	88	28	Chain A, Structure Of The Psedudokinase Domain Of Bir2, An Immune Regulator Of The Rlk/pelle Family
		2nru_A	107	5.00E-26	63	34	Chain A, Crystal Structure Of Irak-4
		2qkw_B	99.4	3.00E-23	69	31	Chain B, Structural Basis For Activation Of Plant Immunity By Bacterial Effector Protein Avrpto
**JM+K+C-term**	**683-1009**	4oh4_A	166	2.00E-47	79	38	Chain A, Crystal Structure Of Bri1 In Complex With Bki1
		3uim_A	130	3.00E-34	69	35	Chain A, Structural Basis For The Impact Of Phosphorylation On Plant Receptor- Like Kinase Bak1 Activation
		4l68_A	108	2.00E-26	99	28	Chain A, Structure Of The Psedudokinase Domain Of Bir2, An Immune Regulator Of The Rlk/pelle Family
		2nru_A	107	2.00E-26	72	34	Chain A, Crystal Structure Of Irak-4
		2qkw_B	99.4	1.00E-23	79	31	Chain B, Structural Basis For

							Activation Of Plant Immunity By Bacterial Effector Protein Avrpto

AA, Amino Acid; Max. Score, Maximum Score; Q C, Query Coverage; Idn., Identity; N-term, N terminal region; LRR, Leucine Rich Repeat; C1, Charged1; TM, Transmembrane; C2, Charged2; JM, Juxtamembrane; K, Kinase; C-term, C terminal.

Table S2B. HHpred analysis of Charged1, Transmembrane and Charged2 regions of the Xa21 protein

**Region**	**AA Boundary**	**PDB ID**	**Score**	**E-value**	**P-value**	**Probability (%)**	**Template Short Identity**
**C1+TM+C2**	**635-682**	2jwa_A	30.7	0.0024	6.70E-08	96.1	Receptor tyrosine-protein kinase ERBB-2
		2ks1_B	27.8	0.016	4.30E-07	95.1	Epidermal growth factor receptor

AA, Amino Acid; C1, Charged1 region; TM, Transmembrane region; C2, Charged 2 region.

Table S3. Predictions of the Leucine rich repeat (LRR) domain and domain boundary using different tools

Domain Name	Song *et al* 1996		HHrepID		lrrfinder.com		SMART	
	**Start AA**	**End AA**	**Start AA**	**End AA**	**Start AA**	**End AA**	**Start AA**	**End AA**
LRR1	81	98	79	101	104	127	102	125
LRR2	99	122	102	125	128	151	126	150
LRR3	123	146	126	149	152	176	151	174
LRR4	147	170	150	172	177	200	199	223
LRR5	172	195	175	198	201	224	247	271
LRR6	196	219	199	222	225	248	320	343
LRR7	220	243	223	246	249*	273*	350	373
LRR8	244	267	247	268	274	321	399	423
LRR9	269	292	272	295	322	351	447	470
LRR10	293	316	296	319	352	376	471	495
LRR11	317	340	320	342	377	400	520	544
LRR12	347	370	350	373	401	448	568	591
LRR13	372	395	376	398	449*	472*		
LRR14	396	419	399	422	473*	545*		
LRR15	420	443	423	446	546	569		
LRR16	444	467	447	470	570	593		
LRR17	468	491	471	494	594**	1025**		
LRR18	492	516	495	519				
LRR19	517	540	520	543				
LRR20	541	564	544	567				
LRR21	565	588	568	591				
LRR22	589	611	592	613				
LRR23	612	634	615	635				

AA, Amino Acid.

*Insignificant hit.

** Potential Leucine Rich Repeat at C terminal.

Table S4. Model validation of different regions of the Xa21 Protein Modeled using the HHpred toolkit

R/P ID	Errat	Verify 3D (%)	Ramachandran Plot Summary from Procheck (%)			
			MFR	AAR	GAR	DR
Xa21lrr_1	67.5	93.91	77.6	19.9	1.3	1.1
Xa21lrr_2	61.333	97.7	72.5	25.2	1.7	0.6
Xa21lrr_3	55	93.59	72.1	24.9	1.1	1.9
Xa21lrr_4	74.167	96.71	76.5	21.8	1.3	0.4
Xa21lrr_5	64.167	96.71	74.6	23.1	1.5	0.8
Xa21lrr_6	61	93.03	71.3	25.8	0.8	2.1
Xa21lrr_7	58.794	96.71	70.6	27.2	1.3	1
Xa21lrr_8	58.667	99.84	71.9	26.4	1	0.8
Xa21lrr_9	71	93.91	75.7	21.2	1.7	1.3
Xa21lrr_10	51.5	95.07	70.7	25.8	1.7	1.7
Xa21lrr_11	61.833	99.67	72.5	1.1	1.1	
Xa21lrr_12	67.667	91.94	75	22.8	1.3	1
Xa21lrr_13	65.5	96.05	71.3	27	0.6	1.1
Xa21lrr_14	59.167	94.9	73.6	24.1	1	1.3
Xa21lrr_15	63.167	100	70.6	26.8	1	1.7
Xa21lrr_16	46.667	91.61	71.5	26.8	1.5	0.2
Xa21lrr_17	61.333	91.78	72.8	25.4	0.8	1
Xa21lrr_18	43.937	84.7	74.8	22.4	2.5	0.4
Xa21lrr_19	59.365	92.93	70.9	27.3	0.8	1
Xa21lrr_20	56.167	95.07	73.6	25.8	1.1	0
Xa21lrr_21	66	96.88	70.7	28.3	0.4	0.6
Xa21lrr_22	61	92.27	71.5	26.8	1	0.8
Xa21lrr_23	58.167	94.41	72.1	26.2	1.5	0.2
Xa21lrr_24	69.5	97.2	75	24.1	0.8	0.2
Xa21lrr_25	60.667	97.86	70.2	27.9	1	1
Xa21lrr_26	62.167	99.34	72.5	26.6	0.8	0.2
Xa21lrr_27	45.74	90.46	69.2	28.3	1.7	0.8
Xa21lrr_28	51.33	93.75	70	27.5	1.5	1

Xa21lrr_29	E	46.05	87	12	0.4	0.6
Xa21lrr_30	56.494	62.17	80.1	17	1.9	1
Xa21lrr_31	72.194	90.95	81.8	16.6	0.6	1
Xa21c12tm_1	67.5	0	95.2	4.8	0	0
Xa21c12tm_2	60.526	0	88.1	11.9	0	0
Xa21c12tm_3	81.579	0	92.9	7.1	0	0
Xa21k_1	80.189	84.71	90.6	7.7	1.4	0.3
Xa21k_2	67.192	74.92	89.2	8	1.7	1
Xa21k_3	50.159	76.45	85	12.9	0.3	1.7
Xa21k_4	64.353	67.89	86	9.4	2.8	1.7
Xa21k_5	67.192	70.03	81.8	15	2.8	0.3
Xa21k_6	70.219	90.52	88.8	7.3	1.4	2.4
Xa21k_7	69.231	82.26	88.8	9.1	1.4	0.7
Xa21k_8	65.696	88.38	87.1	10.5	2.1	0.3
Xa21k_9	69.206	85.93	85.7	10.5	2.1	1.7
Xa21k_10	76.025	80.43	87.8	9.4	2.1	0.7
Xa21k_11	59.873	81.65	88.1	8.7	2.4	0.7
Xa21k_12	59.973	76.15	85	11.9	2.8	0.3
Xa21k_13	73.27	63.91	85.3	12.2	2.1	0.3
Xa21k_14	75.159	82.87	82.5	15	1.7	0.7
Xa21k_15	53.312	66.97	86.4	9.4	2.8	1.4
Xa21k_16	78.797	78.9	87.8	10.8	0.7	0.7
Xa21k_17	74.922	88.07	88.8	9.1	1	1
Xa21k_18	69.401	87.16	88.8	8	2.4	0.7
Xa21k_19	60.568	81.96	87.1	9.8	2.4	0.7
Xa21k_20	67.628	79.51	85	11.2	2.1	1.7
Xa21k_21	66.139	69.72	81.5	15	2.8	0.7
Xa21k_22	77.987	92.97	87.4	10.8	1	0.7
Xa21k_23	67.628	79.51	85	11.2	2.1	1.7
Xa21k_24	62.3	88.38	82.2	16.8	0.3	0.7
Xa21k_25	75.786	85.93	86.4	10.5	1.4	1.7

Xa21k_26	68.671	77.06	88.8	7	3.1	1
Xa21k_27	69.811	86.85	85.7	10.1	2.4	1.7
Xa21k_28	68.889	79.2	82.9	14.7	1.7	0.7
Xa21k_29	74.051	87.16	87.1	10.1	1.4	1.4
Xa21k_30	67.089	89.6	88.8	9.8	0.7	0.7
Xa21k_31	60.417	74.41	87.3	10.4	1.2	1.2

R/P ID, Region or Protein ID; MFR, Most Favoured Regions; AAR, Additionally Allowed Regions; GAR, Generously Allowed Regions; DR, Disallowed Regions; E, Error.

Table S5A. Binding free energy contribution of the key binding-site residues calculated from the binding energy decomposition for Xa21 ($kJmol^{-1}$) of complex 1a and complex 3.

Residues	MM Energy	Polar Energy	Apolar Energy	Total Energy
**GLY-1**	-5.9687 ± 0.0086	0.0015 ± 0.0001	0.0000 ± 0.0000	-5.9673 ± 0.0088
**GLY-1**	-6.6173 ± 0.0110	0.0013 ± 0.0000	0.0000 ± 0.0000	-6.6161 ± 0.0111
**LYS-14**	-7.0653 ± 0.0099	0.0063 ± 0.0001	0.0000 ± 0.0000	-7.0595 ± 0.0100
**LYS-14**	-8.1081 ± 0.0149	0.0017 ± 0.0001	0.0000 ± 0.0000	-8.1064 ± 0.0151
**ARG-46**	-6.0954 ± 0.0079	0.0009 ± 0.0000	0.0000 ± 0.0000	-6.0944 ± 0.0081
**ARG-46**	-6.3763 ± 0.0124	0.0003 ± 0.0000	0.0000 ± 0.0000	-6.3765 ± 0.0119
**ARG-47**	-6.9610 ± 0.0162	-0.0007 ± 0.0000	0.0000 ± 0.0000	-6.9616 ± 0.0169
**ARG-47**	-7.7415 ± 0.0134	0.0016 ± 0.0001	0.0000 ± 0.0000	-7.7399 ± 0.0131
**ARG-48**	-6.8459 ± 0.0124	0.0002 ± 0.0001	0.0000 ± 0.0000	-6.8457 ± 0.0121
**ARG-48**	-6.8279 ± 0.0127	-6.8279 ± 0.0127	0.0000 ± 0.0000	-6.8281 ± 0.0130
**ARG-49**	-5.9652 ± 0.0092	-0.0003 ± 0.0000	0.0000 ± 0.0000	-5.9652 ± 0.0089
**ARG-49**	-6.0932 ± 0.0099	-0.0000 ± 0.0000	0.0000 ± 0.0000	-6.0934 ± 0.0097
**ARG-50**	-5.5553 ± 0.0070	-0.0000 ± 0.0000	0.0000 ± 0.0000	-5.5555 ± 0.0071
**ARG-50**	-5.7137 ± 0.0080	-0.0000 ± 0.0000	0.0000 ± 0.0000	-5.7136 ± 0.0082
**ARG-54**	-6.4122 ± 0.0072	0.0038 ± 0.0001	0.0000 ± 0.0000	-6.4084 ± 0.0071
**ARG-54**	-6.8861 ± 0.0103	0.0038 ± 0.0001	0.0000 ± 0.0000	-6.8820 ± 0.0101
**LYS-57**	-9.0314 ± 0.0137	0.0055 ± 0.0002	0.0000 ± 0.0000	-9.0257 ± 0.0133
**LYS-57**	-8.7035 ± 0.0188	-8.7035 ± 0.0188	0.0000 ± 0.0000	-8.6975 ± 0.0188
**ARG-61**	-11.7671 ± 0.0208	0.0299 ± 0.0009	0.0000 ± 0.0000	-11.7368 ± 0.0211
**ARG-61**	-12.0987 ± 0.0289	0.0007 ± 0.0011	0.0000 ± 0.0000	-12.0966 ± 0.0287
**ARG-80**	-8.1822 ± 0.0129	0.0088 ± 0.0002	0.0000 ± 0.0000	-8.1740 ± 0.0131
**ARG-80**	-7.7939 ± 0.0131	0.0094 ± 0.0003	0.0000 ± 0.0000	-7.7846 ± 0.0129
**ARG-99**	-7.0884 ± 0.0101	0.0123 ± 0.0002	0.0000 ± 0.0000	-7.0757 ± 0.0102
**ARG-99**	-8.1815 ± 0.0156	0.0061 ± 0.0002	0.0000 ± 0.0000	-8.1758 ± 0.0151
**ARG-102**	-6.9704 ± 0.0080	0.0082 ± 0.0001	0.0000 ± 0.0000	-6.9622 ± 0.0080
**ARG-102**	-7.3174 ± 0.0110	0.0070 ± 0.0002	0.0000 ± 0.0000	-7.3110 ± 0.0108
**LYS-126**	-7.7376 ± 0.0109	0.0153 ± 0.0002	0.0000 ± 0.0000	-7.7225 ± 0.0108
**LYS-126**	-7.7596 ± 0.0126	0.0143 ± 0.0004	0.0000 ± 0.0000	-7.7452 ± 0.0123
**ARG-138**	-12.7486 ± 0.0406	0.1348 ± 0.0030	0.0000 ± 0.0000	-12.6158 ± 0.0420
**ARG-138**	-15.9961 ± 0.0589	0.0159 ± 0.0031	0.0000 ± 0.0000	-15.9806 ± 0.0598
**ARG-143**	-9.4608 ± 0.0172	0.0433 ± 0.0010	0.0000 ± 0.0000	-9.4171 ± 0.0167
**ARG-143**	-10.4853 ± 0.0255	0.0920 ± 0.0027	0.0000 ± 0.0000	-10.3915 ± 0.0251
**LYS-150**	-8.6361 ± 0.0148	0.0563 ± 0.0009	0.0000 ± 0.0000	-8.5801 ± 0.0149
**LYS-150**	-8.1667 ± 0.0139	0.0326 ± 0.0007	0.0000 ± 0.0000	-8.1342 ± 0.0140
**LYS-159**	-20.3711 ± 0.0853	0.4720 ± 0.0136	0.0000 ± 0.0000	-19.8979 ± 0.0771
**LYS-159**	-24.6602 ± 0.1174	0.2545 ± 0.0190	0.0000 ± 0.0000	-24.4097 ± 0.1063

**PHE-183**	-0.8145 ± 0.0065	0.3040 ± 0.0067	0.0000 ± 0.0000	-0.5109 ± 0.0066
**PHE-183**	-0.7919 ± 0.0085	0.1788 ± 0.0062	-0.0028 ± 0.0005	-0.6157 ± 0.0064
**ARG-185**	-16.1800 ± 0.1050	0.0717 ± 0.0202	-0.0009 ± 0.0003	-16.1082 ± 0.1047
**ARG-185**	-20.2474 ± 0.1126	0.2793 ± 0.0210	-0.0003 ± 0.0003	-19.9701 ± 0.1071
**ARG-225**	-14.1212 ± 0.0362	0.2711 ± 0.0035	0.0000 ± 0.0000	-13.8508 ± 0.0342
**ARG-225**	-12.4740 ± 0.0454	0.1394 ± 0.0040	0.0000 ± 0.0000	-12.3361 ± 0.0452
**ARG-230**	-51.2735 ± 0.3173	30.8192 ± 0.4278	-0.4258 ± 0.0074	-20.8768 ± 0.1632
**ARG-230**	-45.8491 ± 0.4546	26.7476 ± 0.4994	-0.3229 ± 0.0068	-19.4217 ± 0.1459
**LYS-233**	-25.3704 ± 0.2250	3.8871 ± 0.2018	-0.0159 ± 0.0016	-21.5039 ± 0.1351
**LYS-233**	-19.5911 ± 0.1327	0.9838 ± 0.0358	0.0000 ± 0.0000	-18.6022 ± 0.1167
**LYS-244**	-10.8173 ± 0.0210	0.2516 ± 0.0029	0.0000 ± 0.0000	-10.5651 ± 0.0190
**LYS-244**	-8.7742 ± 0.0230	0.0442 ± 0.0031	0.0000 ± 0.0000	-8.7304 ± 0.0217
**ARG-258**	-23.3446 ± 0.1447	1.6989 ± 0.0749	-0.0021 ± 0.0006	-21.6513 ± 0.1107
**ARG-258**	-16.7439 ± 0.0917	0.5922 ± 0.0170	0.0000 ± 0.0000	-16.1503 ± 0.0838
**LYS-262**	-13.2970 ± 0.0336	0.3120 ± 0.0065	0.0000 ± 0.0000	-12.9841 ± 0.0292
**LYS-262**	-9.8426 ± 0.0371	-0.2366 ± 0.0064	0.0000 ± 0.0000	-10.0802 ± 0.0359
**ARG-293**	-11.5673 ± 0.0262	0.5609 ± 0.0067	0.0000 ± 0.0000	-11.0069 ± 0.0210
**ARG-293**	-8.8897 ± 0.0250	-0.0087 ± 0.0041	0.0000 ± 0.0000	-8.8993 ± 0.0246
**ARG-295**	-11.8382 ± 0.0256	0.5212 ± 0.0068	0.0000 ± 0.0000	-11.3179 ± 0.0217
**ARG-295**	-10.0747 ± 0.0287	0.0856 ± 0.0045	0.0000 ± 0.0000	-9.9901 ± 0.0278
**TRP-303**	-35.7820 ± 0.0836	24.4156 ± 0.0511	-1.9451 ± 0.0072	-1.9451 ± 0.0072
**TRP-303**	-29.0365 ± 0.1575	17.9697 ± 0.1082	-1.3214 ± 0.0084	-12.3897 ± 0.0892
**ARG-304**	-44.8842 ± 0.3680	18.6848 ± 0.4557	-0.6127 ± 0.0094	-26.8115 ± 0.1772
**ARG-304**	-30.1770 ± 0.3256	11.2788 ± 0.4504	-0.4571 ± 0.0105	-19.3743 ± 0.2091
**ARG-310**	-11.7278 ± 0.0279	0.2678 ± 0.0051	0.0000 ± 0.0000	-11.4595 ± 0.0236
**ARG-310**	-8.2929 ± 0.0343	-0.1811 ± 0.0035	0.0000 ± 0.0000	-8.4730 ± 0.0338
**LYS-326**	-14.6779 ± 0.0411	0.7315 ± 0.0092	0.0000 ± 0.0000	-13.9471 ± 0.0348
**LYS-326**	-13.1947 ± 0.0415	0.1141 ± 0.0054	0.0000 ± 0.0000	-13.0792 ± 0.0404
**LYS-368**	-9.2696 ± 0.0292	0.2457 ± 0.0062	0.0000 ± 0.0000	-9.0227 ± 0.0245
**LYS-368**	-8.4412 ± 0.0274	0.0376 ± 0.0032	0.0000 ± 0.0000	-8.4036 ± 0.0249
**TYR-380**	-17.6920 ± 0.1470	12.4566 ± 0.1215	-1.4746 ± 0.0082	-6.7080 ± 0.0751
**TYR-380**	-17.5009 ± 0.1034	13.7472 ± 0.0899	-1.3689 ± 0.0075	-5.1200 ± 0.0786
**ARG-387**	-10.6875 ± 0.0620	0.3596 ± 0.0126	0.0000 ± 0.0000	-10.3284 ± 0.0500
**ARG-387**	-7.1987 ± 0.0565	-0.2713 ± 0.0124	0.0000 ± 0.0000	-7.4707 ± 0.0460
**ARG-396**	-10.3049 ± 0.0263	0.5441 ± 0.0081	0.0000 ± 0.0000	-9.7604 ± 0.0214
**ARG-396**	-11.6806 ± 0.0288	0.5808 ± 0.0069	0.0000 ± 0.0000	-11.0986 ± 0.0258
**LYS-398**	-15.4063 ± 0.0680	1.0771 ± 0.0189	0.0000 ± 0.0000	-14.3305 ± 0.0528
**LYS-398**	-15.6341 ± 0.0597	1.2213 ± 0.0166	0.0000 ± 0.0000	-14.4128 ± 0.0544

**LEU-404**	-5.9956 ± 0.0290	0.0654 ± 0.0063	-0.5861 ± 0.0045	-6.5164 ± 0.0286
**LEU-404**	-7.3450 ± 0.0381	0.1033 ± 0.0064	-0.6953 ± 0.0047	-7.9384 ± 0.0405
**LYS-433**	-8.4102 ± 0.0782	-0.1268 ± 0.0177	0.0000 ± 0.0000	-8.5422 ± 0.0622
**LYS-433**	-5.4489 ± 0.0675	-0.5965 ± 0.0161	0.0000 ± 0.0000	-6.0441 ± 0.0551
**ARG-522**	-5.7350 ± 0.1159	-2.1403 ± 0.0406	-0.0091 ± 0.0014	-7.8838 ± 0.1036
**ARG-522**	-15.9112 ± 0.0918	-0.1566 ± 0.0236	-0.0008 ± 0.0004	-16.0673 ± 0.0839
**PRO-608**	6.1284 ± 0.0213	0.0269 ± 0.0005	0.0000 ± 0.0000	6.1555 ± 0.0216
**PRO-608**	6.9141 ± 0.0275	0.0034 ± 0.0003	0.0000 ± 0.0000	6.9174 ± 0.0278

Green markings represent residues showing higher interaction values; Yellow markings represent residues showing average interaction values.

Table S5B. Binding free energy contribution of the key binding-site residues calculated from the binding energy decomposition for Xa21 ($kJmol^{-1}$) of complex 1b and complex 2.

Residues	MM Energy	Polar Energy	Apolar Energy	Total Energy
**GLY-1**	-9.7687 ± 0.0477	0.0551 ± 0.0374	0.0004 ± 0.0011	-9.7131 ± 0.0613
**GLY-1**	-7.5079 ± 0.0301	0.1413 ± 0.0351	-0.0025 ± 0.0020	-7.3705 ± 0.0457
**LYS-14**	-13.2228 ± 0.0361	0.1253 ± 0.0151	0.0040 ± 0.0023	-13.0940 ± 0.0397
**LYS-14**	-12.7693 ± 0.0555	1.1321 ± 0.0321	0.0015 ± 0.0027	-11.6347 ± 0.0530
**ARG-46**	-8.7174 ± 0.0210	0.0420 ± 0.0153	-0.0010 ± 0.0034	-8.6771 ± 0.0268
**ARG-46**	-7.9087 ± 0.0231	0.0626 ± 0.0178	-0.0027 ± 0.0026	-7.8475 ± 0.0293
**ARG-47**	-10.6854 ± 0.0529	0.0389 ± 0.0182	0.0016 ± 0.0025	-10.6396 ± 0.0568
**ARG-47**	-9.7357 ± 0.0387	0.1075 ± 0.0182	-0.0010 ± 0.0034	-9.6296 ± 0.0430
**ARG-48**	-10.6700 ± 0.0354	0.0453 ± 0.0183	0.0035 ± 0.0029	-10.6213 ± 0.0391
**ARG-48**	-7.3358 ± 0.0293	0.0272 ± 0.0192	0.0024 ± 0.0025	-7.3062 ± 0.0351
**ARG-49**	-8.1130 ± 0.0235	0.0321 ± 0.0192	0.0003 ± 0.0021	-8.0799 ± 0.0299
**ARG-49**	-6.1361 ± 0.0236	0.0446 ± 0.0203	0.0001 ± 0.0017	-6.0928 ± 0.0305
**ARG-50**	-7.2799 ± 0.0188	0.0055 ± 0.0177	-0.0016 ± 0.0017	-7.2757 ± 0.0258
**ARG-50**	-6.1409 ± 0.0224	0.0311 ± 0.0196	0.0030 ± 0.0020	-6.1063 ± 0.0287
**ARG-54**	-9.7234 ± 0.0181	0.0734 ± 0.0167	-0.0057 ± 0.0025	-9.6553 ± 0.0247
**ARG-54**	-8.4683 ± 0.0269	0.1564 ± 0.0186	-0.0045 ± 0.0032	-8.3167 ± 0.0317
**LYS-57**	-15.9923 ± 0.0486	0.1749 ± 0.0320	0.0013 ± 0.0028	-15.8120 ± 0.0575
**LYS-57**	-15.8912 ± 0.0428	0.7352 ± 0.0342	-0.0093 ± 0.0031	-15.1653 ± 0.0514
**ARG-61**	-27.2170 ± 0.0696	1.1002 ± 0.0183	0.0005 ± 0.0032	-26.1161 ± 0.0670
**ARG-61**	-23.5408 ± 0.0673	1.8516 ± 0.0280	-0.0004 ± 0.0021	-21.6908 ± 0.0631
**ARG-80**	-13.1232 ± 0.0303	0.1285 ± 0.0147	-0.0006 ± 0.0023	-12.9947 ± 0.0328
**ARG-80**	-10.9371 ± 0.0295	0.3749 ± 0.0153	-0.0028 ± 0.0026	-10.5658 ± 0.0311
**ARG-99**	-12.8909 ± 0.0278	0.1415 ± 0.0157	0.0030 ± 0.0022	-12.7473 ± 0.0316
**ARG-99**	-8.8306 ± 0.0444	0.1862 ± 0.0175	0.0012 ± 0.0028	-8.6446 ± 0.0450
**ARG-102**	-10.9864 ± 0.0197	0.1216 ± 0.0162	-0.0030 ± 0.0022	-10.8691 ± 0.0253
**ARG-102**	-10.7391 ± 0.0396	0.3819 ± 0.0176	-0.0023 ± 0.0027	-10.3588 ± 0.0387

**LYS-126**	-12.0785 ± 0.0219	0.1093 ± 0.0350	-0.0044 ± 0.0026	-11.9721 ± 0.0409
**LYS-126**	-10.7144 ± 0.0278	0.2339 ± 0.0350	-0.0017 ± 0.0027	-10.4822 ± 0.0420
**ARG-138**	-60.8613 ± 0.3245	12.7899 ± 0.3309	-0.4855 ± 0.0125	-48.5570 ± 0.1529
**ARG-138**	-49.5846 ± 0.5068	22.8854 ± 0.4595	-0.9228 ± 0.0164	-27.6154 ± 0.2646
**ARG-143**	-16.7600 ± 0.0606	0.0757 ± 0.0182	0.0025 ± 0.0019	-16.6808 ± 0.0643
**ARG-143**	-13.3313 ± 0.0719	0.4140 ± 0.0186	-0.0012 ± 0.0029	-12.9194 ± 0.0750
**LYS-150**	-13.0782 ± 0.0282	0.1741 ± 0.0336	-0.0002 ± 0.0025	-12.9043 ± 0.0425
**LYS-150**	-9.8829 ± 0.0297	0.2478 ± 0.0338	-0.0023 ± 0.0023	-9.6369 ± 0.0439
**LYS-159**	-73.7898 ± 0.3612	54.5355 ± 0.6430	-1.1879 ± 0.0091	-20.4579 ± 0.3622
**LYS-159**	-91.6969 ± 0.3070	75.9152 ± 0.6604	-1.7192 ± 0.0106	-17.4810 ± 0.4501
**PHE-183**	-13.6334 ± 0.0724	4.4258 ± 0.0718	-2.2752 ± 0.0075	-11.4786 ± 0.0552
**PHE-183**	-15.3290 ± 0.0619	8.0958 ± 0.0418	-2.0325 ± 0.0088	-9.2657 ± 0.0619
**ARG-185**	-113.5355 ± 0.5004	88.6535 ± 0.4657	-2.6790 ± 0.0145	-27.5459 ± 0.2336
**ARG-185**	-74.7709 ± 0.4661	58.1120 ± 0.6605	-1.8893 ± 0.0163	-18.5529 ± 0.2538
**ARG-225**	-17.6968 ± 0.0623	-0.0072 ± 0.0153	-0.0004 ± 0.0023	-17.7024 ± 0.0654
**ARG-225**	-17.9356 ± 0.0651	0.1791 ± 0.0164	0.0061 ± 0.0034	-17.7503 ± 0.0659
**ARG-230**	-37.4765 ± 0.2447	4.4832 ± 0.2574	-0.1962 ± 0.0080	-33.1992 ± 0.1140
**ARG-230**	-39.5601 ± 0.1824	9.1658 ± 0.1995	-0.1078 ± 0.0049	-30.5009 ± 0.1469
**LYS-233**	-31.9923 ± 0.2901	2.9629 ± 0.2473	-0.0813 ± 0.0046	-29.1132 ± 0.1918
**LYS-233**	-19.4239 ± 0.1366	0.3268 ± 0.0549	-0.0026 ± 0.0021	-19.0988 ± 0.1230
**LYS-244**	-9.1315 ± 0.0192	0.0687 ± 0.0366	-0.0037 ± 0.0027	-9.0665 ± 0.0431
**LYS-244**	-7.7724 ± 0.0322	0.0699 ± 0.0368	-0.0010 ± 0.0031	-7.7030 ± 0.0477
**ARG-258**	-17.3387 ± 0.1255	-0.3142 ± 0.0374	-0.0019 ± 0.0027	-17.6526 ± 0.1205
**ARG-258**	-16.1880 ± 0.1022	0.3972 ± 0.0434	-0.0033 ± 0.0024	-15.7961 ± 0.0937
**LYS-262**	-8.0191 ± 0.0282	0.0078 ± 0.0362	-0.0031 ± 0.0029	-8.0134 ± 0.0471
**LYS-262**	-6.8243 ± 0.0348	0.0015 ± 0.0369	-0.0012 ± 0.0038	-6.8230 ± 0.0501
**ARG-293**	-8.1288 ± 0.0219	0.0178 ± 0.0148	-0.0036 ± 0.0028	-8.1146 ± 0.0271
**ARG-293**	-7.4879 ± 0.0309	-0.0379 ± 0.0152	0.0026 ± 0.0038	-7.5227 ± 0.0319
**ARG-295**	-9.0904 ± 0.0235	0.0002 ± 0.0156	0.0001 ± 0.0023	-9.0921 ± 0.0280
**ARG-295**	-9.5394 ± 0.0308	0.0043 ± 0.0176	0.0037 ± 0.0029	-9.5315 ± 0.0350
**TRP-303**	-0.8300 ± 0.0061	-0.0050 ± 0.0070	-0.0006 ± 0.0024	-0.8357 ± 0.0100
**TRP-303**	0.2776 ± 0.0173	-0.2211 ± 0.0131	0.0018 ± 0.0024	0.0580 ± 0.0133
**ARG-304**	-6.4413 ± 0.1145	-0.5703 ± 0.0485	-0.0017 ± 0.0020	-7.0067 ± 0.1053
**ARG-304**	-9.9707 ± 0.0927	-0.3307 ± 0.0196	0.0001 ± 0.0024	-10.2977 ± 0.0899
**ARG-310**	-6.3695 ± 0.0226	0.0136 ± 0.0165	-0.0007 ± 0.0027	-6.3562 ± 0.0276
**ARG-310**	-6.0378 ± 0.0236	0.0127 ± 0.0155	0.0068 ± 0.0029	-6.0196 ± 0.0280
**LYS-326**	-12.4760 ± 0.0391	0.0166 ± 0.0333	-0.0036 ± 0.0026	-12.4629 ± 0.0522
**LYS-326**	-14.4390 ± 0.0570	-0.0933 ± 0.0350	-0.0013 ± 0.0029	-14.5316 ± 0.0653

**LYS-368**	-4.9048 ± 0.0218	0.0424 ± 0.0357	0.0012 ± 0.0020	-4.8615 ± 0.0419
**LYS-368**	-5.6806 ± 0.0319	0.0640 ± 0.0339	-0.0016 ± 0.0026	-5.6164 ± 0.0459
**TYR-380**	-0.0417 ± 0.0044	0.0071 ± 0.0093	0.0010 ± 0.0025	-0.0333 ± 0.0109
**TYR-380**	-1.1177 ± 0.0377	0.6878 ± 0.0470	-0.0535 ± 0.0035	-0.4825 ± 0.0217
**ARG-387**	-3.6936 ± 0.0291	0.0355 ± 0.0161	0.0004 ± 0.0022	-3.6592 ± 0.0338
**ARG-387**	-4.2876 ± 0.0465	-0.0777 ± 0.0161	0.0009 ± 0.0021	-4.3656 ± 0.0470
**ARG-396**	-7.1893 ± 0.0237	0.0282 ± 0.0166	0.0009 ± 0.0026	-7.1616 ± 0.0294
**ARG-396**	-7.7624 ± 0.0312	-0.0491 ± 0.0173	0.0049 ± 0.0028	-7.8072 ± 0.0351
**LYS-398**	-11.9927 ± 0.0495	0.0646 ± 0.0333	-0.0044 ± 0.0024	-11.9305 ± 0.0580
**LYS-398**	-9.1156 ± 0.0365	-9.1156 ± 0.0365	0.0052 ± 0.0025	-9.1836 ± 0.0489
**LEU-404**	-0.1325 ± 0.0014	-0.0008 ± 0.0024	0.0015 ± 0.0024	-0.1319 ± 0.0037
**LEU-404**	-0.5346 ± 0.0064	0.0512 ± 0.0027	-0.0045 ± 0.0027	-0.4880 ± 0.0061
**LYS-433**	-2.7775 ± 0.0368	0.0212 ± 0.0322	0.0031 ± 0.0026	-2.7561 ± 0.0494
**LYS-433**	-3.7705 ± 0.0586	0.0964 ± 0.0254	-0.0013 ± 0.0017	-3.6767 ± 0.0637
**ARG-522**	-26.6830 ± 0.3555	16.4826 ± 0.4122	-0.6542 ± 0.0112	-10.8558 ± 0.1752
**ARG-522**	-72.0310 ± 0.5148	73.5158 ± 0.5014	-1.9091 ± 0.0136	-0.4415 ± 0.3213
**PRO-608**	-22.3819 ± 0.4220	4.0357 ± 0.2917	-0.0727 ± 0.0058	-18.4335 ± 0.2748
**PRO-608**	-4.4437 ± 0.1578	0.2507 ± 0.0439	0.0002 ± 0.0024	-4.1905 ± 0.1637

Green markings represent residues showing higher interaction values; Yellow markings represent residues showing average interaction values.

Table S5C. Binding free energy contribution of the key binding-site residues calculated from the binding energy decomposition for RaxX21-sY ($kJmol^{-1}$) of complex 1a and complex 3.

Residues	MM Energy	Polar Energy	Apolar Energy	Total Energy
**VAL-2**	-24.6045 ± 0.1332	12.6492 ± 0.1220	-1.5783 ± 0.0093	-13.5344 ± 0.0765
**VAL-2**	-13.0105 ± 0.1020	7.0837 ± 0.0886	-0.5546 ± 0.0079	-6.4884 ± 0.0514
**GLY-3**	-13.6600 ± 0.1274	8.9606 ± 0.1514	-1.4675 ± 0.0076	-6.1731 ± 0.0591
**GLY-3**	-16.0877 ± 0.1713	12.1984 ± 0.1730	-1.0387 ± 0.0132	-4.9325 ± 0.0554
**PRO-14**	-13.3340 ± 0.0830	5.3404 ± 0.0618	-1.2992 ± 0.0104	-9.2938 ± 0.0723
**PRO-14**	-18.7857 ± 0.1110	7.9902 ± 0.0944	-1.8124 ± 0.0082	-12.6051 ± 0.0563
**LYS-15**	-88.6216 ± 0.4083	81.5238 ± 0.7059	-2.6593 ± 0.0128	-9.7518 ± 0.4931
**LYS-15**	-65.9948 ± 0.3908	53.6579 ± 0.7919	-2.0346 ± 0.0109	-14.3603 ± 0.5324

Green markings represent residues showing higher interaction values; Yellow markings represent residues showing average interaction values.

Table S5D. Binding free energy contribution of the key binding-site residues calculated from the binding energy decomposition for OsSERK2 (kJmol^{-1}) of complex 1b and complex 2.

Residues	MM Energy	Polar Energy	Apolar Energy	Total Energy
**LYS-46**	20.2439 ± 0.2959	-0.6592 ± 0.1510	-0.0167 ± 0.0039	19.5717 ± 0.2614
**LYS-46**	-15.7543 ± 0.3780	3.4063 ± 0.2380	-0.1518 ± 0.0063	-12.4987 ± 0.2876
**ASP-47**	-47.8659 ± 0.2763	22.9919 ± 0.2677	-0.0552 ± 0.0039	-24.9359 ± 0.1862
**ASP-47**	-16.3727 ± 0.2586	0.0132 ± 0.1916	-0.0054 ± 0.0031	-16.3583 ± 0.1525
**LEU-59**	-8.3827 ± 0.0711	0.0982 ± 0.0334	-1.2386 ± 0.0090	-9.5206 ± 0.0585
**LEU-59**	-4.8219 ± 0.0675	0.1678 ± 0.0182	-0.5323 ± 0.0122	-5.1878 ± 0.0849
**ASP-80**	-23.5021 ± 0.2628	5.3938 ± 0.1197	0.0047 ± 0.0045	-18.1082 ± 0.1873
**ASP-80**	-40.5096 ± 0.2140	10.5007 ± 0.1642	-0.0024 ± 0.0043	-30.0019 ± 0.1448
**PHE-137**	-13.6270 ± 0.0961	4.9343 ± 0.0622	-1.6782 ± 0.0128	-10.3732 ± 0.0657
**PHE-137**	-24.0051 ± 0.2385	14.2520 ± 0.1632	-1.4993 ± 0.0222	-11.2639 ± 0.1178
**LYS-164**	-85.3566 ± 0.6218	63.6532 ± 0.9761	-0.6962 ± 0.0122	-22.4333 ± 0.4886
**LYS-164**	-53.8833 ± 0.6652	28.6518 ± 0.7936	-0.6757 ± 0.0153	-25.9628 ± 0.3910
**LYS-183**	-56.9942 ± 0.4190	6.9810 ± 0.2642	-0.0728 ± 0.0063	-50.0972 ± 0.2914
**LYS-183**	-42.8270 ± 0.4131	10.7269 ± 0.4008	-0.1961 ± 0.0093	-32.3350 ± 0.2338
**ARG-192**	-18.2370 ± 0.1072	0.9081 ± 0.0294	-0.0042 ± 0.0049	-17.3383 ± 0.1031
**ARG-192**	-18.2371 ± 0.2376	1.3801 ± 0.0571	-0.0050 ± 0.0048	-16.8526 ± 0.2047
**ARG-212**	-51.5250 ± 0.4645	8.0290 ± 0.3351	-0.2861 ± 0.0124	-43.7914 ± 0.2648
**ARG-212**	-34.6025 ± 0.3026	2.0019 ± 0.0980	-0.0222 ± 0.0051	-32.6356 ± 0.2480
**ARG-218**	-14.9573 ± 0.1097	0.5054 ± 0.0249	-0.0056 ± 0.0045	-14.4520 ± 0.1065
**ARG-218**	-15.4568 ± 0.1678	0.6392 ± 0.0271	-0.0000 ± 0.0050	-14.8206 ± 0.1576
**LYS-222**	-8.8753 ± 0.0885	0.3428 ± 0.0542	0.0018 ± 0.0043	-8.5338 ± 0.1009
**LYS-222**	-7.5329 ± 0.1065	0.5910 ± 0.0484	0.0029 ± 0.0041	-6.9419 ± 0.1145
**LYS-232**	-6.4713 ± 0.0955	0.2985 ± 0.0473	-0.0015 ± 0.0053	-6.1758 ± 0.1048
**LYS-232**	-0.0178 ± 0.0810	0.5993 ± 0.0478	0.0012 ± 0.0059	0.5819 ± 0.0915
**LYS-238**	-11.9570 ± 0.1207	0.1138 ± 0.0472	-0.0023 ± 0.0047	-11.8461 ± 0.1287
**LYS-238**	-6.7402 ± 0.1094	0.3987 ± 0.0503	0.0018 ± 0.0052	-6.3363 ± 0.1185

Green markings represent residues showing higher interaction values; Yellow markings represent residues showing average interaction values.

Table S6A: Protein-Protein Main Chain-Side Chain Hydrogen Bonds of complex 1.

Before Simulation								
Protein-Protein Main Chain-Side Chain Hydrogen Bonds								
DONOR				ACCEPTOR				
**POS**	**CHAIN**	**RES**	**ATOM**	**POS**	**CHAIN**	**RES**	**ATOM**	**Dd-a**
**62**	A	SER	OG	72	C	PRO	O	3.18
**86**	A	ASP	OD2	71	C	ASN	O	3.46
**86**	A	ASP	OD2	71	C	ASN	O	3.46
**110**	A	ASP	OD2	63	C	CYS	O	2.78
**110**	A	ASP	OD2	63	C	CYS	O	2.78
**134**	A	HIS	NE2	63	C	CYS	O	3.19
**134**	A	HIS	ND1	64	C	THR	O	3.17
**134**	A	HIS	ND1	65	C	TRP	O	2.68
**134**	A	HIS	NE2	65	C	TRP	O	2.05
**134**	A	HIS	NE2	68	C	VAL	O	3.08
**159**	A	LYS	NZ	65	C	TRP	O	3.4
**159**	A	LYS	NZ	66	C	PHE	O	3.37
**207**	A	GLN	OE1	53	C	GLN	O	2.01
**207**	A	GLN	OE1	53	C	GLN	O	2.01
**331**	**A**	**ASN**	**ND2**	1	B	HIS	O	2.33
**331**	**A**	**ASN**	**ND2**	1	B	HIS	O	2.33
**567**	**A**	**THR**	**N**	**140**	**C**	**GLU**	**OE1**	3.34
**567**	**A**	**THR**	**N**	**140**	**C**	**GLU**	**OE2**	1.63
**568**	**A**	**MET**	**N**	**140**	**C**	**GLU**	**OE2**	3
**4**	B	GLY	N	301	A	TYR	OH	3.4
**11**	B	GLY	N	93	C	GLN	NE2	2.59
**16**	B	HIS	NE2	43	C	GLN	O	2.5
**21**	B	ARG	N	39	C	TYR	OH	3.03
**21**	B	ARG	N	43	C	GLN	OE1	2.25
**21**	B	ARG	N	43	C	GLN	NE2	2.89
**36**	C	ASP	OD1	8	B	PRO	O	3.26
**36**	C	ASP	OD1	8	B	PRO	O	3.26
**39**	C	TYR	OH	6	B	ASP	O	1.86
**40**	C	SER	OG	10	B	PRO	O	2.06
**42**	C	ARG	NE	21	B	ARG	O	2.91
**43**	C	GLN	NE2	6	B	ASP	O	3.34
**43**	C	GLN	NE2	6	B	ASP	O	3.34

**43**	C	GLN	OE1	21	B	ARG	O	3.09
**43**	C	GLN	OE1	21	B	ARG	O	3.09
**43**	C	GLN	OE1	21	B	ARG	OXT	3.19
**43**	C	GLN	OE1	21	B	ARG	OXT	3.19
**65**	C	TRP	N	134	A	HIS	NE2	2.92
**66**	C	PHE	N	134	A	HIS	ND1	3.36
**116**	C	ASN	ND2	544	A	GLY	O	2.47
**116**	C	ASN	ND2	544	A	GLY	O	2.47
**140**	C	GLU	OE1	565	A	ASP	O	3.12
**140**	C	GLU	OE1	565	A	ASP	O	3.12
**140**	C	GLU	OE2	565	A	ASP	O	3.5
**140**	C	GLU	OE2	565	A	ASP	O	3.5
**After Simulation**								
**Protein-Protein Main Chain-Side Chain Hydrogen Bonds**								
**DONOR**				**ACCEPTOR**				
**POS**	**CHAIN**	**RES**	**ATOM**	**POS**	**CHAIN**	**RES**	**ATOM**	**Dd-a**
**185**	A	ARG	NH2	52	C	LEU	O	3.36
**185**	A	ARG	NH2	52	C	LEU	O	3.36
**303**	A	TRP	NE1	1	B	HIS	O	3.06
**303**	A	TRP	NE1	4	B	GLY	O	3.00
**331**	**A**	**ASN**	**ND2**	2	B	VAL	O	2.97
**331**	**A**	**ASN**	**ND2**	2	B	VAL	O	2.97
**382**	A	CYS	SG	15	B	LYS	O	3.90
**544**	A	GLY	N	116	C	ASN	ND2	3.43
**567**	**A**	**THR**	**N**	**140**	**C**	**GLU**	**OE1**	3.10
**567**	**A**	**THR**	**N**	**140**	**C**	**GLU**	**OE2**	2.91
**568**	**A**	**MET**	**N**	**140**	**C**	**GLU**	**OE1**	2.94
**1**	B	HIS	N	334	A	GLU	OE1	3.06
**1**	B	HIS	N	334	A	GLU	OE2	3.38
**1**	B	HIS	N	358	A	GLU	OE2	2.94
**2**	B	VAL	N	358	A	GLU	OE2	3.43
**6**	B	ASP	N	279	A	TYR	OH	2.73
**53**	C	GLN	NE2	183	A	PHE	O	2.84
**53**	C	GLN	NE2	183	A	PHE	O	2.84
**53**	C	GLN	OE1	184	A	ASN	O	3.07
**53**	C	GLN	OE1	184	A	ASN	O	3.07

Pos, Position; Res, Residue; Dd-a, Distance between donor atom and acceptor atom; Red colored residues are hot residues; Green color indicates sustained bonds after simulation; Bold residues are sustained residues after simulation.

Table S 6b: Protein-Protein Side Chain-Side Chain Hydrogen Bonds from complex 1

Before Simulation								
Protein-Protein Side Chain-Side Chain Hydrogen Bonds								
DONOR				ACCEPTOR				
**POS**	**CHAIN**	**RES**	**ATOM**	**POS**	**CHAIN**	**RES**	**ATOM**	**Dd-a**
**207**	A	GLN	OE1	53	C	GLN	NE2	2.83
**207**	A	GLN	OE1	53	C	GLN	NE2	2.83
**230**	A	ARG	NH2	56	C	ASP	OD2	2.5
**230**	**A**	**ARG**	**NH2**	56	C	ASP	OD2	2.5
**231**	A	GLU	OE1	53	C	GLN	OE1	3.09
**231**	A	GLU	OE1	53	C	GLN	OE1	3.09
**231**	A	GLU	OE1	53	C	GLN	NE2	2.7
**231**	A	GLU	OE1	53	C	GLN	NE2	2.7
**231**	A	GLU	OE2	53	C	GLN	OE1	1.69
**231**	A	GLU	OE2	53	C	GLN	OE1	1.69
**231**	A	GLU	OE2	53	C	GLN	NE2	2.24
**231**	A	GLU	OE2	53	C	GLN	NE2	2.24
**279**	A	TYR	OH	6	B	ASP	OD1	3.34
**279**	A	TYR	OH	6	B	ASP	OD2	3.15
**358**	A	GLU	OE1	1	B	HIS	ND1	3.06
**358**	A	GLU	OE1	1	B	HIS	ND1	3.06
**522**	A	ARG	NH1	117	C	GLU	OE1	2.98
**522**	A	ARG	NH1	117	C	GLU	OE1	2.98
**546**	A	GLU	OE2	116	C	ASN	ND2	3.28
**546**	A	GLU	OE2	116	C	ASN	ND2	3.28
**567**	**A**	**THR**	**OG1**	**140**	**C**	**GLU**	**OE1**	2.36
**567**	A	THR	OG1	140	C	GLU	OE2	2.27
**1**	B	HIS	ND1	358	A	GLU	OE1	3.06
**15**	B	LYS	NZ	383	A	ASN	OD1	2.99
**15**	B	LYS	NZ	383	A	ASN	ND2	2.63
**21**	B	ARG	NH2	231	A	GLU	OE1	2.1
**21**	B	ARG	NH2	231	A	GLU	OE1	2.1
**21**	B	ARG	NH1	279	A	TYR	OH	3.38
**21**	B	ARG	NH1	279	A	TYR	OH	3.38
**53**	C	GLN	NE2	207	A	GLN	OE1	2.83
**53**	C	GLN	NE2	207	A	GLN	OE1	2.83
**53**	C	GLN	OE1	231	A	GLU	OE1	3.09
**53**	C	GLN	OE1	231	A	GLU	OE1	3.09

**53**	C	GLN	OE1	231	A	GLU	OE2	1.69
**53**	C	GLN	OE1	231	A	GLU	OE2	1.69
**53**	C	GLN	NE2	231	A	GLU	OE1	2.7
**53**	C	GLN	NE2	231	A	GLU	OE1	2.7
**53**	C	GLN	NE2	231	A	GLU	OE2	2.24
**53**	C	GLN	NE2	231	A	GLU	OE2	2.24
**113**	C	THR	OG1	546	A	GLU	OE1	3.48
**116**	C	ASN	ND2	546	A	GLU	OE2	3.28
**116**	C	ASN	ND2	546	A	GLU	OE2	3.28
**116**	C	ASN	OD1	568	A	MET	SD	1.15
**116**	C	ASN	OD1	568	A	MET	SD	1.15
**116**	C	ASN	ND2	568	A	MET	SD	2.66
**116**	C	ASN	ND2	568	A	MET	SD	2.66
**After Simulation**								
**Protein-Protein Side Chain-Side Chain Hydrogen Bonds**								
**DONOR**				**ACCEPTOR**				
**POS**	**CHAIN**	**RES**	**ATOM**	**POS**	**CHAIN**	**RES**	**ATOM**	**Dd-a**
**209**	A	ASN	ND2	50	C	ASN	OD1	2.83
**209**	A	ASN	ND2	50	C	ASN	OD1	2.83
**230**	**A**	**ARG**	**NH2**	6	B	ASP	OD1	3.44
**230**	A	ARG	NH2	6	B	ASP	OD1	3.44
**406**	A	TYR	OH	16	B	HIS	NE2	2.78
**543**	A	LYS	NZ	117	C	GLU	OE1	3.17
**543**	A	LYS	NZ	117	C	GLU	OE2	3.37
**567**	**A**	**THR**	**OG1**	**140**	**C**	**GLU**	**OE2**	2.85
**16**	B	HIS	NE2	406	A	TYR	OH	2.78
**50**	C	ASN	OD1	209	A	ASN	ND2	2.83
**50**	C	ASN	OD1	209	A	ASN	ND2	2.83

Pos, Position; Res, Residue; Dd-a, Distance between donor atom and acceptor atom; Red colored residues are hot residues; Green color indicates sustained bonds after simulation; Bold residues are sustained residues after simulation.

Table S6C: Protein-Protein Hydrophobic Interactions from complex 1

**Before Simulation**					
**Protein-Protein Hydrophobic Interactions**					
**Position**	**Residue**	**Chain**	**Position**	**Residue**	**Chain**
**8**	PRO	B	39	TYR	C
**9**	PRO	B	31	LEU	C
**88**	TYR	A	77	ILE	C
**183**	**PHE**	**A**	**59**	**LEU**	**C**

**183**	PHE	A	65	TRP	C
**183**	PHE	A	66	PHE	C
**301**	TYR	A	2	VAL	B
**303**	**TRP**	**A**	**19**	**PRO**	**B**
**354**	PHE	A	2	VAL	B
**380**	TYR	A	2	VAL	B
**404**	LEU	A	14	PRO	B
**426**	ILE	A	14	PRO	B
**428**	**LEU**	**A**	**14**	**PRO**	**B**
**568**	**MET**	**A**	**139**	**PRO**	**C**
**569**	LEU	A	137	PHE	C
**588**	ALA	A	137	PHE	C
**591**	ALA	A	137	PHE	C
**After Simulation**					
**Protein-Protein Hydrophobic Interactions**					
**Position**	**Residue**	**Chain**	**Position**	**Residue**	**Chain**
**183**	**PHE**	**A**	**59**	**LEU**	**C**
**303**	**TRP**	**A**	**19**	**PRO**	**B**
**356**	ALA	A	2	VAL	B
**380**	TYR	A	14	PRO	B
**380**	TYR	A	2	VAL	B
**402**	ILE	A	14	PRO	B
**428**	**LEU**	**A**	**14**	**PRO**	**B**
**568**	**MET**	**A**	**139**	**PRO**	**C**

Red colored residues are hot residues; Green color indicates sustain bond after simulation.

Table S6D: Protein-Protein Ionic Interactions from complex 1

**Before Simulation**					
**Protein-Protein Ionic Interactions**					
**Position**	**Residue**	**Chain**	**Position**	**Residue**	**Chain**
**230**	ARG	A	56	ASP	C
**230**	**ARG**	**A**	**6**	**ASP**	**B**
**231**	**GLU**	**A**	**21**	**ARG**	**B**
**231**	GLU	A	42	ARG	C
**253**	ASP	A	21	ARG	B
**304**	ARG	A	6	ASP	B
**334**	**GLU**	**A**	**1**	**HIS**	**B**
**358**	GLU	A	1	HIS	B
**358**	GLU	A	15	LYS	B

**407**	GLU	A	15	LYS	B
**522**	ARG	A	117	GLU	C
**543**	**LYS**	**A**	140	GLU	C
**565**	**ASP**	**A**	**164**	**LYS**	**C**
**After Simulation**					
**Protein-Protein Ionic Interactions**					
**Position**	**Residue**	**Chain**	**Position**	**Residue**	**Chain**
**21**	ARG	B	46	LYS	C
**185**	ARG	A	47	ASP	C
**230**	**ARG**	**A**	**6**	**ASP**	**B**
**231**	**GLU**	**A**	**21**	**ARG**	**B**
**304**	ARG	A	17	ASP	B
**334**	**GLU**	**A**	**1**	**HIS**	**B**
**334**	GLU	A	16	HIS	B
**543**	**LYS**	**A**	117	GLU	C
**565**	**ASP**	**A**	**164**	**LYS**	**C**

Red colored residues are hot residues; Green color indicates sustained bonds after simulation; Bold residues are sustained residues after simulation.

Table S6E: Cation-Pi Interactions within 6 Angstroms from complex 1.

**Before Simulation**							
**Cation-Pi Interactions within 6 Angstroms**							
**Position**	**Residue**	**Chain**	**Position**	**Residue**	**Chain**	**D (cation-pi)**	**Angle**
**66**	**PHE**	**C**	159	LYS	A	4.06	121.59
**279**	TYR	A	21	ARG	B	5.75	75.47
**After Simulation**							
**Cation-Pi Interactions within 6 Angstroms**							
**Position**	**Residue**	**Chain**	**Position**	**Residue**	**Chain**	**D (cation-pi)**	**Angle**
**66**	**PHE**	**C**	138	ARG	A	4.21	17.57
**406**	TYR	A	15	LYS	B	5.96	148.06

D (cation-pi**)**, Distance between two chains; Red colored residues are hot residues; Bold residues are sustained residues after simulation.

Table S6F: Protein-Protein Aromatic-Sulphur Interactions from complex 1

Before Simulation							
**Protein-Protein Aromatic-Sulphur Interactions**							
**NO PROTEIN-PROTEIN AROMATIC-SULPHUR INTERACTIONS FOUND**							
**After Simulation**							
**Protein-Protein Aromatic-Sulphur Interactions**							
**Position**	**Residue**	**Chain**	**Position**	**Residue**	**Chain**	**D(Centroid-Sulphur)**	**Angle**
**137**	PHE	C	568	MET	A	5.10	52.38

D(Centroid-Sulphur), Distance between two chains

Table S6G: Protein-Protein Main Chain-Main Chain Hydrogen Bonds from complex 2

Before Simulation								
**Protein-Protein Main Chain-Main Chain Hydrogen Bonds**								
**No Protein-Protein Main Chain-Main Chain Hydrogen Bonds**								
**After Simulation**								
**Protein-Protein Main Chain-Main Chain Hydrogen Bonds**								
**DONOR**				**ACCEPTOR**				
**POS**	**CHAIN**	**RES**	**ATOM**	**POS**	**CHAIN**	**RES**	**ATOM**	**Dd-a**
**164**	C	LYS	N	567	A	THR	O	3.11

Pos, Position; Res, Residue; Dd-a, Distance between donor atom and acceptor atom; Red colored residues are hot residues.

Table S6H: Protein-Protein Main Chain-Side Chain Hydrogen Bonds from complex 2

Before Simulation								
**Protein-Protein Main Chain-Side Chain Hydrogen Bonds**								
**DONOR**				**ACCEPTOR**				
**POS**	**CHAIN**	**RES**	**ATOM**	**POS**	**CHAIN**	**RES**	**ATOM**	**Dd-a**
**62**	A	SER	OG	72	C	PRO	O	3.18
**86**	A	ASP	OD2	71	C	ASN	O	3.46
**86**	A	ASP	OD2	71	C	ASN	O	3.46
**110**	A	ASP	OD2	63	C	CYS	O	2.78
**110**	A	ASP	OD2	63	C	CYS	O	2.78
**134**	A	HIS	NE2	63	C	CYS	O	3.19
**134**	A	HIS	ND1	64	C	THR	O	3.17
**134**	A	HIS	ND1	65	C	TRP	O	2.68
**134**	A	HIS	NE2	65	C	TRP	O	2.05
**134**	A	HIS	NE2	68	C	VAL	O	3.08
**159**	A	LYS	NZ	65	C	TRP	O	3.4

**159**	A	LYS	NZ	66	C	PHE	O	3.37
**207**	A	GLN	OE1	53	C	GLN	O	2.01
**207**	A	GLN	OE1	53	C	GLN	O	2.01
**567**	A	THR	N	140	C	GLU	OE1	3.34
**567**	A	THR	N	140	C	GLU	OE2	1.63
**568**	A	MET	N	140	C	GLU	OE2	3
**65**	C	TRP	N	134	A	HIS	NE2	2.92
**66**	C	PHE	N	134	A	HIS	ND1	3.36
**116**	C	ASN	ND2	544	A	GLY	O	2.47
**116**	C	ASN	ND2	544	A	GLY	O	2.47
**140**	C	GLU	OE1	565	A	ASP	O	3.12
**140**	C	GLU	OE1	565	A	ASP	O	3.12
**140**	C	GLU	OE2	565	A	ASP	O	3.5
**140**	C	GLU	OE2	565	A	ASP	O	3.5
				**After Simulation**				
				**Protein-Protein Main Chain-Side Chain Hydrogen Bonds**				
		**DONOR**				**ACCEPTOR**		
**POS**	**CHAIN**	**RES**	**ATOM**	**POS**	**CHAIN**	**RES**	**ATOM**	**Dd-a**
**522**	A	ARG	NE	114	C	ILE	O	3.4
**522**	A	ARG	NH2	115	C	PRO	O	3.02
**522**	A	ARG	NH2	115	C	PRO	O	3.02
**522**	A	ARG	NH1	135	C	THR	O	3
**522**	A	ARG	NH1	135	C	THR	O	3
**522**	A	ARG	NH1	137	C	PHE	O	3.04
**522**	A	ARG	NH1	137	C	PHE	O	3.04
**522**	A	ARG	NH2	137	C	PHE	O	3.44
**522**	A	ARG	NH2	137	C	PHE	O	3.44
**50**	C	ASN	N	231	A	GLU	OE2	2.94
**137**	C	PHE	N	546	A	GLU	OE2	2.91
**161**	C	SER	OG	569	A	LEU	O	3.47
**161**	C	SER	N	570	A	HIS	ND1	3.06

Pos, Position; Res, Residue; Dd-a, Distance between donor atom and acceptor atom; Red colored residues are hot residues.

Table S6I: Protein-Protein Side Chain-Side Chain Hydrogen Bonds from complex 2

				**Before Simulation**				
				**Protein-Protein Side Chain-Side Chain Hydrogen Bonds**				
		**DONOR**				**ACCEPTOR**		
**POS**	**CHAIN**	**RES**	**ATOM**	**POS**	**CHAIN**	**RES**	**ATOM**	**Dd-a**

**207**	A	GLN	OE1	53	C	GLN	NE2	2.83
**207**	A	GLN	OE1	53	C	GLN	NE2	2.83
**230**	A	ARG	NH2	56	C	ASP	OD2	2.5
**230**	A	ARG	NH2	56	C	ASP	OD2	2.5
**231**	**A**	**GLU**	**OE1**	53	C	GLN	OE1	3.09
**231**	**A**	**GLU**	**OE1**	53	C	GLN	OE1	3.09
**231**	**A**	**GLU**	**OE1**	53	C	GLN	NE2	2.7
**231**	**A**	**GLU**	**OE1**	53	C	GLN	NE2	2.7
**231**	A	GLU	OE2	53	C	GLN	OE1	1.69
**231**	A	GLU	OE2	53	C	GLN	OE1	1.69
**231**	A	GLU	OE2	53	C	GLN	NE2	2.24
**231**	A	GLU	OE2	53	C	GLN	NE2	2.24
**522**	**A**	**ARG**	**NH1**	117	C	GLU	OE1	2.98
**522**	**A**	**ARG**	**NH1**	117	C	GLU	OE1	2.98
**546**	A	GLU	OE2	116	C	ASN	ND2	3.28
**546**	A	GLU	OE2	116	C	ASN	ND2	3.28
**567**	A	THR	OG1	140	C	GLU	OE1	2.36
**567**	**A**	**THR**	**OG1**	**140**	**C**	**GLU**	**OE2**	2.27
**53**	C	GLN	NE2	207	A	GLN	OE1	2.83
**53**	C	GLN	NE2	207	A	GLN	OE1	2.83
**53**	C	GLN	OE1	231	A	GLU	OE1	3.09
**53**	C	GLN	OE1	231	A	GLU	OE1	3.09
**53**	C	GLN	OE1	231	A	GLU	OE2	1.69
**53**	C	GLN	OE1	231	A	GLU	OE2	1.69
**53**	C	GLN	NE2	231	A	GLU	OE1	2.7
**53**	C	GLN	NE2	231	A	GLU	OE1	2.7
**53**	C	GLN	NE2	231	A	GLU	OE2	2.24
**53**	C	GLN	NE2	231	A	GLU	OE2	2.24
**113**	C	THR	OG1	546	A	GLU	OE1	3.48
**116**	C	ASN	ND2	546	A	GLU	OE2	3.28
**116**	C	ASN	ND2	546	A	GLU	OE2	3.28
**116**	**C**	**ASN**	**OD1**	**568**	**A**	**MET**	**SD**	1.15
**116**	**C**	**ASN**	**OD1**	**568**	**A**	**MET**	**SD**	1.15
**116**	**C**	**ASN**	**ND2**	**568**	**A**	**MET**	**SD**	2.66
**116**	**C**	**ASN**	**ND2**	**568**	**A**	**MET**	**SD**	2.66
				**After Simulation**				
				**Protein-Protein Side Chain-Side Chain Hydrogen Bonds**				
		**DONOR**				**ACCEPTOR**		

POS	CHAIN	RES	ATOM	POS	CHAIN	RES	ATOM	Dd-a
**88**	A	TYR	OH	73	C	ASP	OD1	2.8
**136**	A	GLN	OE1	61	C	ASN	ND2	3.13
**136**	A	GLN	OE1	61	C	ASN	ND2	3.13
**136**	A	GLN	NE2	61	C	ASN	ND2	3.27
**136**	A	GLN	NE2	61	C	ASN	ND2	3.27
**138**	A	ARG	NH1	71	C	ASN	OD1	3.04
**138**	A	ARG	NH1	71	C	ASN	OD1	3.04
**185**	A	ARG	NH2	50	C	ASN	OD1	3.37
**185**	A	ARG	NH2	50	C	ASN	OD1	3.37
**185**	A	ARG	NH2	67	C	HIS	ND1	2.83
**185**	A	ARG	NH2	67	C	HIS	ND1	2.83
**231**	**A**	**GLU**	**OE1**	49	C	ASN	ND2	3.01
**231**	**A**	**GLU**	**OE1**	49	C	ASN	ND2	3.01
**231**	**A**	**GLU**	**OE1**	50	C	ASN	ND2	2.99
**231**	**A**	**GLU**	**OE1**	50	C	ASN	ND2	2.99
**495**	A	LYS	NZ	93	C	GLN	OE1	2.85
**496**	A	ASN	ND2	93	C	GLN	OE1	2.94
**496**	A	ASN	ND2	93	C	GLN	OE1	2.94
**522**	**A**	**ARG**	**NE**	116	C	ASN	OD1	2.69
**522**	**A**	**ARG**	**NH2**	116	C	ASN	OD1	3.03
**522**	A	ARG	NH2	116	C	ASN	OD1	3.03
**567**	**A**	**THR**	**OG1**	**140**	**C**	**GLU**	**OE2**	2.73
**570**	A	HIS	ND1	161	C	SER	OG	3.44
**49**	C	ASN	ND2	231	A	GLU	OE1	3.01
**49**	C	ASN	ND2	231	A	GLU	OE1	3.01
**50**	C	ASN	ND2	231	A	GLU	OE1	2.99
**50**	C	ASN	ND2	231	A	GLU	OE1	2.99
**61**	C	ASN	ND2	136	A	GLN	OE1	3.13
**61**	C	ASN	ND2	136	A	GLN	OE1	3.13
**61**	C	ASN	ND2	136	A	GLN	NE2	3.27
**61**	C	ASN	ND2	136	A	GLN	NE2	3.27
**93**	C	GLN	OE1	496	A	ASN	ND2	2.94
**93**	C	GLN	OE1	496	A	ASN	ND2	2.94
**116**	**C**	**ASN**	**OD1**	**568**	**A**	**MET**	**SD**	3.68
**116**	**C**	**ASN**	**OD1**	**568**	**A**	**MET**	**SD**	3.68
**116**	**C**	**ASN**	**ND2**	**568**	**A**	**MET**	**SD**	3.32

**116**	C	ASN	ND2	568	A	MET	SD	3.32
**161**	C	SER	OG	570	A	HIS	ND1	3.44

Pos, Position; Res, Residue; Dd-a, Distance between donor atom and acceptor atom; Red colored residues are hot residues; Green color indicates sustained bonds after simulation; Bold residues are sustained residues after simulation.

Table S6J: Protein-Protein Hydrophobic Interactions from complex 2

**Before Simulation**					
**Protein-Protein Hydrophobic Interactions**					
**Position**	**Residue**	**Chain**	**Position**	**Residue**	**Chain**
**88**	TYR	A	77	ILE	C
**183**	PHE	A	59	LEU	C
**183**	PHE	A	65	TRP	C
**183**	PHE	A	66	PHE	C
**568**	**MET**	**A**	139	PRO	C
**569**	LEU	A	137	PHE	C
**588**	ALA	A	137	PHE	C
**591**	ALA	A	137	PHE	C
**After Simulation**					
**Protein-Protein Hydrophobic Interactions**					
**Position**	**Residue**	**Chain**	**Position**	**Residue**	**Chain**
**183**	PHE	A	66	PHE	C
**474**	ILE	A	91	VAL	C
**520**	LEU	A	115	PRO	C
**568**	**MET**	**A**	137	PHE	C
**568**	MET	A	163	PRO	C

Bold residues are sustained residues after simulation.

Table S6K: Protein-Protein Ionic Interactions from complex 2

**Before Simulation**					
**Protein-Protein Ionic Interactions**					
**Position**	**Residue**	**Chain**	**Position**	**Residue**	**Chain**
**230**	ARG	A	56	ASP	C
**231**	GLU	A	42	ARG	C
**522**	ARG	A	117	GLU	C
**543**	**LYS**	**A**	140	GLU	C
**565**	ASP	A	164	LYS	C
**After Simulation**					
**Protein-Protein Ionic Interactions**					
**Position**	**Residue**	**Chain**	**Position**	**Residue**	**Chain**
**159**	LYS	A	56	ASP	C
**543**	**LYS**	**A**	117	GLU	C

Red coloured residues are hot residues; Bold residues are sustained residues after simulation.

Table S6L: Protein-Protein Cation-Pi Interactions from complex 2

Before Simulation							
Protein-Protein Cation-Pi Interactions							
**Position**	**Residue**	**Chain**	**Position**	**Residue**	**Chain**	**D(cation-Pi)**	**Angle**
**66**	**PHE**	**C**	159	LYS	A	4.06	121.59
**After Simulation**							
**Protein-Protein Cation-Pi Interactions**							
**Position**	**Residue**	**Chain**	**Position**	**Residue**	**Chain**	**D(cation-Pi)**	**Angle**
**66**	**PHE**	**C**	185	ARG	A	3.89	39.7

D (cation-pi**)**, Distance between two chains; Red colored residues are hot residues; Bold residues are sustained residues after simulation.

Table S6M: Protein-Protein Aromatic-Aromatic Interactions from complex 2

Before Simulation							
**Protein-Protein Aromatic-Aromatic Interactions**							
**NO PROTEIN-PROTEIN AROMATIC-AROMATIC INTERACTIONS FOUND**							
**After Simulation**							
**Protein-Protein Aromatic-Aromatic Interactions**							
**Position**	**Residue**	**Chain**	**Position**	**Residue**	**Chain**	**D(cation-Pi)**	**Angle**
**183**	PHE	A	66	PHE	C	5.77	51.71

D(cation-pi**)**: Distance between two chains

Table S6N: Protein-Protein Main Chain-Side Chain Hydrogen Bonds from complex 3

Before Simulation								
**Protein-Protein Main Chain-Side Chain Hydrogen Bonds**								
	**DONOR**				**ACCEPTOR**			
**POS**	**CHAIN**	**RES**	**ATOM**	**POS**	**CHAIN**	**RES**	**ATOM**	**Dd-a**
**331**	**A**	**ASN**	**ND2**	**1**	**B**	**HIS**	**O**	2.33
**331**	**A**	**ASN**	**ND2**	**1**	**B**	**HIS**	**O**	2.33
**4**	B	GLY	N	301	A	TYR	OH	3.4
**After Simulation**								
**Protein-Protein Main Chain-Side Chain Hydrogen Bonds**								
	**DONOR**				**ACCEPTOR**			
**POS**	**CHAIN**	**RES**	**ATOM**	**POS**	**CHAIN**	**RES**	**ATOM**	**Dd-a**
**303**	A	TRP	NE1	1	B	HIS	O	3.27
**331**	**A**	**ASN**	**ND2**	**1**	**B**	**HIS**	**O**	3.47
**331**	**A**	**ASN**	**ND2**	**1**	**B**	**HIS**	**O**	3.47
**331**	A	ASN	ND2	2	B	VAL	O	3.08

**331**	A	ASN	ND2	2	B	VAL	O	3.08
**382**	A	CYS	SG	14	B	PRO	O	3.55
**1**	B	HIS	N	334	A	GLU	OE1	3.38
**1**	B	HIS	N	334	A	GLU	OE2	3.33
**1**	B	HIS	N	358	A	GLU	OE2	2.95
**4**	B	GLY	N	331	A	ASN	OD1	3.41
**5**	B	GLY	N	301	A	TYR	OH	2.72

Pos, Position; Res, Residue; Dd-a, Distance between donor atom and acceptor atom; Red colored residues are hot residues; Green color indicates sustained bonds after simulation; Bold residues are sustained residues after simulation.

Table S6O: Protein-Protein Side Chain-Side Chain Hydrogen Bonds from complex 3

**Before Simulation**								
**Protein-Protein Side Chain-Side Chain Hydrogen Bonds**								
**DONOR**				**ACCEPTOR**				
**POS**	**CHAIN**	**RES**	**ATOM**	**POS**	**CHAIN**	**RES**	**ATOM**	**Dd-a**
**279**	A	TYR	OH	6	B	ASP	OD1	3.34
**279**	A	TYR	OH	6	B	ASP	OD2	3.15
**358**	A	GLU	OE1	1	B	HIS	ND1	3.06
**358**	A	GLU	OE1	1	B	HIS	ND1	3.06
**1**	B	HIS	ND1	358	A	GLU	OE1	3.06
**7**	**B**	**X**	**OH**	380	A	TYR	OH	3.07
**15**	B	LYS	NZ	383	A	ASN	OD1	2.99
**15**	B	LYS	NZ	383	A	ASN	ND2	2.63
**21**	B	ARG	NH2	231	A	GLU	OE1	2.1
**21**	B	ARG	NH2	231	A	GLU	OE1	2.1
**21**	B	ARG	NH1	279	A	TYR	OH	3.38
**21**	B	ARG	NH1	279	A	TYR	OH	3.38
**After Simulation**								
**Protein-Protein Side Chain-Side Chain Hydrogen Bonds**								
**DONOR**				**ACCEPTOR**				
**POS**	**CHAIN**	**RES**	**ATOM**	**POS**	**CHAIN**	**RES**	**ATOM**	**Dd-a**
**378**	A	HIS	NE2	7	B	X	OH	2.83
**7**	**B**	**X**	**OH**	378	A	HIS	NE2	2.83

Pos, Position; Res, Residue; Dd-a, Distance between donor atom and acceptor atom; Red colored residues are hot residues; Green color indicates sustained bonds after simulation; Bold residues are sustained residues after simulation.

Table S6P: Protein-Protein Hydrophobic Interactions from complex 3

**Before Simulation**					
**Protein-Protein Hydrophobic Interactions**					
**Position**	**Residue**	**Chain**	**Position**	**Residue**	**Chain**
**301**	TYR	A	2	VAL	B
**303**	TRP	A	19	PRO	B

**354**	PHE	A	2	VAL	B
**380**	**TYR**	**A**	**2**	**VAL**	**B**
**404**	**LEU**	**A**	**14**	**PRO**	**B**
**426**	ILE	A	14	PRO	B
**428**	LEU	A	14	PRO	B
**After Simulation**					
**Protein-Protein Hydrophobic Interactions**					
**Position**	**Residue**	**Chain**	**Position**	**Residue**	**Chain**
**380**	TYR	A	14	PRO	B
**380**	**TYR**	**A**	**2**	**VAL**	**B**
**402**	ILE	A	12	ALA	B
**404**	**LEU**	**A**	**14**	**PRO**	**B**

Red colored residues are hot residues; Green color indicates sustain bond after simulation.

Table S6Q: Protein-Protein Ionic Interactions from complex 3

**Before Simulation**					
**Protein-Protein Ionic Interactions**					
**Position**	**Residue**	**Chain**	**Position**	**Residue**	**Chain**
**230**	**ARG**	**A**	**6**	**ASP**	**B**
**231**	GLU	A	21	ARG	B
**253**	**ASP**	**A**	21	ARG	B
**304**	**ARG**	**A**	6	ASP	B
**334**	**GLU**	**A**	**1**	**HIS**	**B**
**358**	GLU	A	1	HIS	B
**358**	GLU	A	15	LYS	B
**407**	**GLU**	**A**	15	LYS	B
**After Simulation**					
**Protein-Protein Ionic Interactions**					
**Position**	**Residue**	**Chain**	**Position**	**Residue**	**Chain**
**230**	**ARG**	**A**	**6**	**ASP**	**B**
**304**	**ARG**	**A**	17	ASP	B
**334**	**GLU**	**A**	**1**	**HIS**	**B**
**407**	**GLU**	**A**	16	HIS	B

Red colored residues are hot residues; Green color indicates sustained bonds after simulation; Bold residues are sustained residues after simulation.

Table S6R: Protein-Protein Cation-Pi Interactions from complex 3

**Before Simulation**							
**Protein-Protein Cation-Pi Interactions**							
**Position**	**Residue**	**Chain**	**Position**	**Residue**	**Chain**	**D(cation-Pi)**	**Angle**
**279**	TYR	A	21	ARG	B	5.75	75.47

After Simulation
**Protein-Protein Cation-Pi Interactions**
**NO PROTEIN-PROTEIN CATION-PI INTERACTIONS FOUND**

D (cation-pi**)**, Distance between two chain

Complex	Inter. Bet.	H-Bond		Hydrophobic Interaction		Ionic Interaction		Cation - Pi Interaction		Aromatic - Aromatic Interaction		Aromatic - Sulphar Interaction		Total	
		B. MD	*A. MD*	*B. MD*	*A. MD*	*B. MD*	*A. MD*	*B. MD*	*A. MD*	*B. MD*	*A. MD*	*B. MD*	*A. MD*	*B. MD*	*A. MD*
**Xa21 +Raxsn +Os**	*Xa21 +Raxsn*	15	14	7	6	8	5	1	1	0	0	0	0	31	26
	Xa21 +Os	60	17	8	2	5	3	1	1	0	0	0	1	74	24
	Raxsn +Os	16	0	2	0	0	1	0	0	0	0	0	0	18	1
**Xa21 +Os**	*Xa21 +Os*	60	52	8	5	5	2	1	1	0	1	0	0	74	79
**Xa21 +Raxsn**	*Xa21 +Raxsn*	15	13	7	4	8	4	1	0	0	0	0	0	31	21

Table S6S: Summary of interactions between Xa21, RaxX21-sY and OsSERK2

Inter. Bet., Interactions Between; H-Bond, Hydrogen Bond; B. MD, Before Molecular Dynamics simulation; A. MD, After Molecular Dynamics simulation
